# Transcriptome integrated metabolic modeling of carbon assimilation underlying storage root development in cassava

**DOI:** 10.1038/s41598-021-88129-3

**Published:** 2021-04-22

**Authors:** Ratchaprapa Kamsen, Saowalak Kalapanulak, Porntip Chiewchankaset, Treenut Saithong

**Affiliations:** 1 Bioinformatics and Systems Biology Program, School of Bioresources and Technology and School of Information Technology, King Mongkut’s University of Technology Thonburi (Bang Khun Thian), Bangkok, 10150 Thailand; 2 Center for Agricultural Systems Biology, Systems Biology and Bioinformatics Research Group, Pilot Plant Development and Training Institute, King Mongkut’s University of Technology Thonburi (Bang Khun Thian), Bangkok, 10150 Thailand

**Keywords:** Biochemical networks, Metabolic engineering, Computer modelling, Systems biology, Computational biology and bioinformatics, Biochemical reaction networks, Computational models, Data integration, Metabolic engineering

## Abstract

The existing genome-scale metabolic model of carbon metabolism in cassava storage roots, rMeCBM, has proven particularly resourceful in exploring the metabolic basis for the phenotypic differences between high and low-yield cassava cultivars. However, experimental validation of predicted metabolic fluxes by carbon labeling is quite challenging. Here, we incorporated gene expression data of developing storage roots into the basic flux-balance model to minimize infeasible metabolic fluxes, denoted as rMeCBMx, thereby improving the plausibility of the simulation and predictive power. Three different conceptual algorithms, GIMME, E-Flux, and HPCOF were evaluated. The rMeCBMx-HPCOF model outperformed others in predicting carbon fluxes in the metabolism of storage roots and, in particular, was highly consistent with transcriptome of high-yield cultivars. The flux prediction was improved through the oxidative pentose phosphate pathway in cytosol, as has been reported in various studies on root metabolism, but hardly captured by simple FBA models. Moreover, the presence of fluxes through cytosolic glycolysis and alanine biosynthesis pathways were predicted with high consistency with gene expression levels. This study sheds light on the importance of prediction power in the modeling of complex plant metabolism. Integration of multi-omics data would further help mitigate the ill-posed problem of constraint-based modeling, allowing more realistic simulation.

## Introduction

Carbon assimilation is an essential metabolic process underlying the biosynthesis of basic building blocks for organismal growth and development. In plants, the process is related to the conversion of atmospheric carbon dioxide captured during photosynthesis to carbon-derived compounds composed of cellular biomass^1^. Carbon metabolism is highly conserved across species and, even, kingdoms. Comprehension of its complexity has remained elusive^2,3^. Despite similarities in carbon assimilation pathways in plants, there is an abundance of diverse metabolic products varying in quantity. In addition to the sophisticated and highly redundant relationships between metabolic reactions and metabolites, the process dynamically changes depending on prevailing conditions. Isotope labeling is typically used to gain insights into the metabolic conversion of carbon^4^; nonetheless, its application in plants is challenging due to expensive instrumentation, the short half-life of labeled precursors, and heterogeneity of plant cells, i.e. multicellular organisms with diverse cellular compartments^5,6^, among others.

Genome-scale metabolic models (GSMMs) have been introduced to complement experimental studies^7^ and enhance our understanding of complex metabolic pathways in plants. They enable interpolation of flux conversion for metabolic intermediates between the measurable biomolecules, connecting metabolic phenotypes to the physiological growth of plants. GSMMs are basically constructed from the entire metabolic reactions annotated for any studied genome^8^ and simulated according to the thermodynamic mass balance and condition-specific constraints, so-called constraint-based modeling (CBM)^9^. The first model of the minimal sets of metabolic reactions required for plant growth under heterotrophic conditions was developed for Arabidopsis^10^. Genome-wide metabolic models exploring primary and secondary cellular metabolisms in plants, e.g. AraGEM^11^, as well as models enabling the study of tissue-level (leaves, roots, seeds, flowers, etc.) metabolism^12^ have also been developed. Moreover, CBM has deepened our understanding of metabolism, yield improvement and stress response in major crop plants, for example, changes in metabolic behavior in response to drought and flooding in rice^13^, lipid biosynthesis and accumulation in developing embryos of rapeseed^14^, and metabolism underlying photosynthesis, photorespiration, and respiration in maize^15^. During the past year, CBM was used to study carbon assimilation in cassava storage roots (rMeCBM)^16^ through a simple flux balance analysis (FBA). The rMeCBM model demonstrated cultivar-dependent differences in carbon utilization for storage root development, particularly between KU50 (Kasetsart 50), a high-yield cultivar, and HN (Hanatee), a low-yield cultivar. In addition, rMeCBM described the growth phenotype of cassava storage roots and robustly predicted carbon assimilation in both cultivars, but direct validation of the interpolated intracellular fluxes was experimentally infeasible. Performing a C-labeling experiment with tuber plants such as cassava remains a challenge.

Given their simplicity and minimal data requirement, FBA models often weakly predict metabolic fluxes at elaborate branched-chain reactions and in a cyclic pathway, for example, pentose-phosphate pathway and non-cyclic TCA, where fluxes change dynamically with prevailing environments^17–19^. Hence, omics data is incorporated into the model to improve the prediction quality, thereby making the model context-specific^20,21^. According to the central dogma of gene to protein function, metabolic reactions cannot achieve their function unless the enzymatic protein-coding genes are expressed. Transcriptome data, which provide genome-wide gene expression information, are then exploited to trace the active reactions associated with metabolic processes under specific conditions. Covert et al. proposed the first transcriptome-integrated CBM, investigated metabolic fluxes in central carbon metabolism of *Escherichia coli* (*E. coli*)*,* and demonstrated the effect of transcriptional regulation on metabolism^22^. Various computational methods have been developed to constrain inactive metabolic reactions under context-specific conditions during the last decades. Among them, GIMME (Gene Inactivity Moderated by Metabolism and Expression) pre-defines inactive reactions based on a low-level of enzymatic gene expression under a set threshold and then minimizes fluxes through these reactions. The method incorporates gene expression to constrain the CBM model as demonstrated for *E. coli*^23^ and in Arabidopsis leaves under drought stress^24^. To avoid the bias of threshold setting, E-Flux was proposed to directly constrain predicted fluxes to the measured level of gene expression. This method was originally developed to study changes in metabolic flux capacity in *Mycobacterium tuberculosis*^25^, and Arabidopsis rosettes^26^. Later, Lee et al. proposed a method to maximize the correlation between the magnitude of reaction fluxes and expression levels of corresponding enzymatic genes^27^. The HPCOF (Huber Penalty Convex Optimization Function) approach uses the Huber penalty function to resolve local optima problems in the previous approaches. The method was successfully applied to study metabolic fluxes in both *E. coli* and yeast metabolism^28^. Although gene expression data do not directly relate to fluxes because of post-transcriptional and translational modifications, they can confine the viable flux space to reflect the biological behavior better than the generic CBM model^26^. To date, very few studies have incorporated transcriptome data into CBM models of plants^24,26,29,30^, especially plant storage organs.

Here, transcriptome-integrated CBMs (rMeCBMx) were developed to improve metabolic flux prediction of carbon assimilation in cassava storage roots (rMeCBM). The models were constructed based on transcriptome data of developing storage roots^31^, using three different conceptual methods, denoted as rMeCBMx-GIMME, rMeCBMx-EFlux, and rMeCBMx-HPCOF. The performance of the models was analyzed through the consistency of the predicted fluxes and expression of corresponding genes in various datasets of cassava storage roots. Results showed the prediction by rMeCBMx-HPCOF corresponded best to the metabolic gene expression observed in cassava storage roots, especially in the high-yield cultivar. Compared to the original rMeCBM model, rMeCBMx-HPCOF showed a significant improvement in carbon flux prediction in the complicated branched pathways: (1) carbon substrates supplied via oxidative pentose phosphate pathway, (2) TCA cycle and glycolysis in respiration pathway, and (3) carbon precursors for alanine biosynthesis. These reactions were consistently weakly predicted by the basic FBA model. This study showed that integration of gene expression data could enhance the prediction power of the rMeCBM model, yielding more biologically relevant information on carbon assimilation in developing storage roots of cassava.

## Methods

### Transcriptome data analysis of cassava storage roots

Eight RNA-seq datasets of developing storage roots of cassava were collected from the literature^31,32^. The transcriptome data included expression of 33,033 genes in developing storage roots of four cassava cultivars (Supplementary Table S1). Expression data of 762 metabolic genes associated with biochemical reactions in rMeCBM were analyzed by Cufflinks^33^, and then we calculated the fragments per kilobase of transcript per million mapped reads (FPKM)^34^. The rMeCBMx models were constrained using gene expression data from Wilson et al.^31^, which contained the highest number of replications among available transcriptome datasets.

The predictive performance of the models was assessed by comparing the metabolic fluxes with the expressed enzymatic genes in three representative scenarios: (1) in developing storage roots of cassava (eight RNA-seq datasets from Wang et al.^32^; Wilson et al.^31^; scenario 1), (2) in developing storage roots of high-yield cultivars (six datasets of KU50 and Arg7 from Wang et al.^32^; scenario 2), and (3) in developing storage roots of the KU50 cassava cultivar (three datasets from Wang et al.^32^; scenario 3). A meta-transcriptome analysis of datasets in each scenario was performed, and only concurrently expressed genes across datasets were collected to represent genes expressed in the condition. Genes were considered similarly expressed between datasets under similar conditions (same scenario) if the coefficient of variation (CV) of expression across datasets was less than 25 percent.

### Transcriptome-integrated constraint-based metabolic models of cassava storage roots

#### rMeCBMx

**Figure 1:**
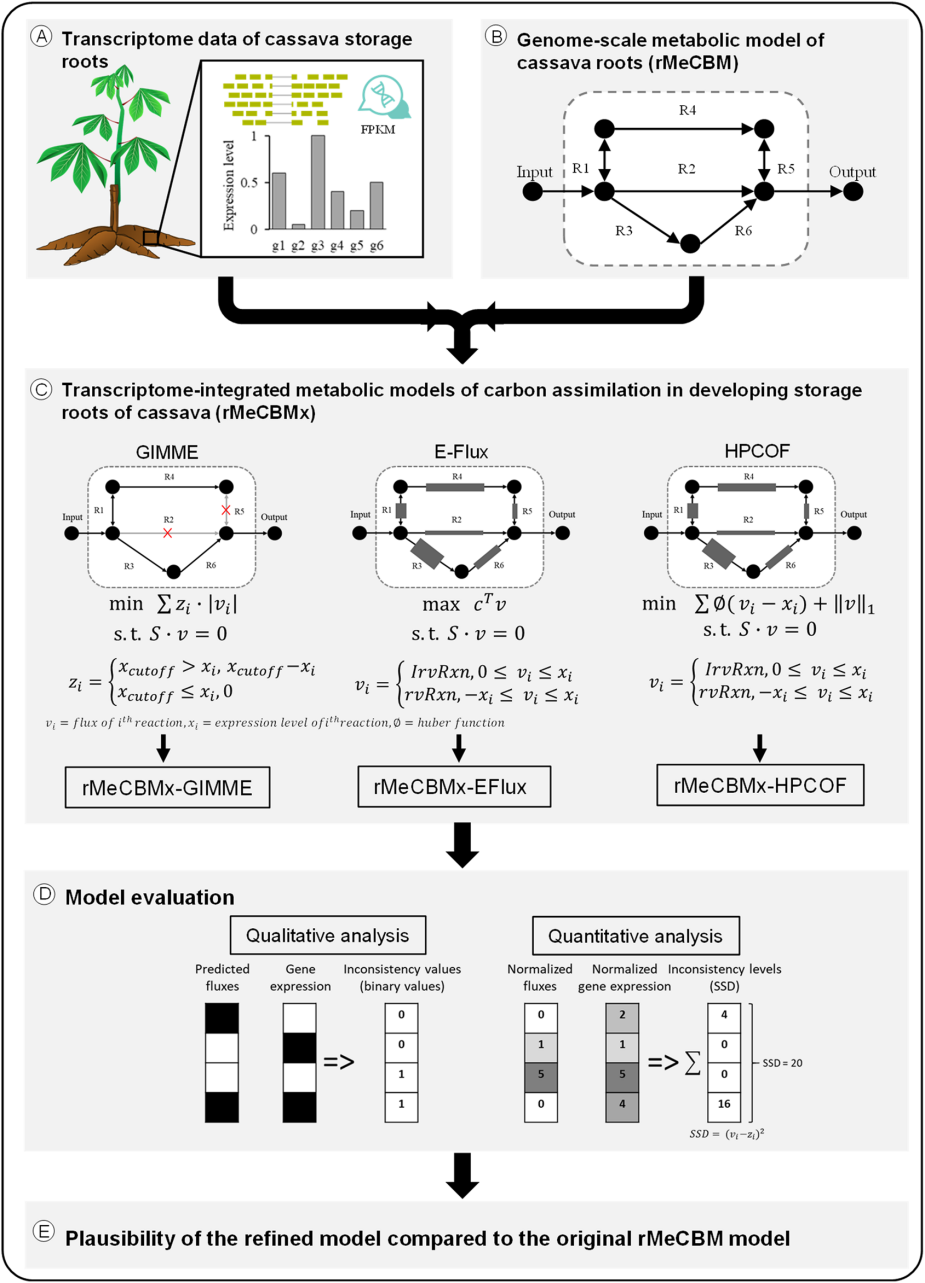
Overall methodology. (**A**) Transcriptome data analysis of gene expression in developing storage roots of cassava. (**B**) Genome-scale metabolic model of cassava storage roots, rMeCBM, obtained from Chiewchankaset et al*.*^16^. (**C**) Development of transcriptome-constrained GSMM models through GIMME, E-Flux, and HPCOF methods. Metabolic fluxes of each reaction were constrained by the relative expression level of the highest expressed genes responsible for the reaction. (**D**) Evaluation of model performance based on the consistency of predicted fluxes with gene expression levels, both qualitatively and quantitatively. (**E**) Plausibility testing of the transcriptome-constrained GSMM model by simulation of storage root growth of the high-yielding cultivar CMC-9, sensitivity analysis, and analysis of the consistency of predicted flux distribution with the gene expression data.

The genome-scale metabolic model of cassava storage roots (rMeCBM) by Chiewchankaset et al.^16^ formed the basis of this study. The rMeCBM model covers seven pathways: the starch and sucrose biosynthesis pathway (SSP), respiration pathway (RES), pentose-phosphate pathway (PPP), cell wall biosynthesis pathway (CEL), amino acid biosynthesis pathway (AMI), fatty acid biosynthesis pathway (FAT), and nucleotide biosynthesis pathway (NUC), essentially representing carbon assimilation toward the biosynthesis of storage root biomass. It contains 393 metabolites and 468 reactions, namely 330 biochemical reactions associated with 762 enzymatic genes in cassava genome version 6.1, 116 transport and exchange reactions, and 22 auxiliary and non-gene reactions. In this study, the model of carbon assimilation in developing storage roots of cassava was simulated according to growth and physiological data of the KU50 cassava cultivar as presented in the original publication (specific sucrose uptake rate to roots = 0.0548 mmol_Suc_gDW^−^1_storage roots_ day^−1^ and storage root growth rate = 0.0090 day^−1^)^16^. To refine the prediction of metabolic conversion in storage roots, the rMeCBM model was further constrained with high-resolution gene expression data of developing storage roots of cassava^31^, denoted as rMeCBMx (Fig. 1). The transcriptome-integrated constraint-based models were constructed by three methods, i.e. GIMME, E-Flux, HPCOF, and named accordingly: rMeCBMx-GIMME, rMeCBMx-EFlux, and rMeCBMx-HPCOF. The rMeCBMx-GIMME and rMeCBMx-EFlux were carried out using COBRA Toolbox 2.0.5^35^, while rMeCBMx-HPCOF was performed using HPCOF algorithms^28^ with CVX solver (version 2.0)^36,37^. All models were simulated in MATLAB (The Math Works, version R2019b) environment.

#### rMeCBMx-GIMME

As guided by gene expression levels, GIMME^23^ minimized predicted fluxes through inactive reactions in the metabolism of developing storage roots (Eq. 1). The inactive reactions were defined by the weak expression of responsible genes relative to set thresholds, which herein were the 25th (low-rank), 50^th^ (middle-rank), and 75^th^ (high-rank) percentiles of all enzymatic gene expression in the rMeCBM model.

1 $$Minimize \mathop \sum \limits_{j = 1}^{n} c_{j} \cdot \left| {v_{j} } \right|.$$

Subject to

$$\mathop \sum \limits_{j = 1}^{n} S_{ij} v_{j} = 0,\,\,\forall \, \,metabolite \,i,$$

$$v_{j,min} < v_{j} < v_{j, max} , \,\,\forall \,\, reaction \,j,$$

$$c_{j} = \left\{ {x_{threshold} - x_{j} \,where \,x_{threshold} > x_{j} , \,0 \,otherwise} \right\},$$

where $S_{ij}$ is the stoichiometric coefficient of metabolite *i* participating in reaction *j*; $v_{j}$ represents flux through reaction j; $v_{j,min}$ and $v_{j,max}$ respectively represent lower and upper bounds of the flux through reaction *j*; $x_{j}$ is the normalized expression level of genes associated with reaction *j*; $x_{threshold}$ is the gene expression threshold, and $c_{j}$ is the penalty score.

#### rMeCBMx-EFlux

Here, it was assumed that the gene expression level determined the activity of enzymes in each metabolic reaction. The capacity of fluxes was estimated based on the abundance of enzymatic gene expression^25^. The rMeCBMx-EFlux model employed information on the gene expression level (*x*) to set the boundary of flux for each reaction (*j*), enabling the optimization of biomass production to simulate the growth of cassava storage roots (Eq. 2). In particular, the gene expression data was normalized by percentile rank to reconcile the range of expression between datasets and then employed to constrain the reaction fluxes by scaling to the original viable space of the model. The boundary of flux solution was set to constraint-free for non-gene-associated reactions.

2 $${\text{Maximize}}\,\,v_{biomass} .$$

Subject to

$$\mathop \sum \limits_{j = 1}^{n} S_{ij} v_{j} = 0,\,\forall \,\,metabolite \,i,$$

$$0 \le v_{j} \le x_{j} , \,\forall \,\, irrevesible \,reaction \,j,$$

$$- x_{j} \le v_{j} \le x_{j} , \,\forall \,\,revesible\, reaction \,j.$$

#### rMeCBMx-HPCOF

The HPCOF model^28^ minimizes the distance between the predicted fluxes ($$v_{j}$$) and gene expression levels ($$x_{j}$$) using the Huber penalty function ($$\emptyset \left( u \right)$$), exploiting the ability of the $$\ell_{1}$$-norm regularization ($$ IIvII_1$$) to perform feature selection (Eq. 3). The Huber penalty function is a robust penalty convex function that confers a unique solution for global optimization.

3 $$\text{Minimize}~ \sum \limits_{j = 1}^{n} \emptyset \left( {v_{j} - x_{j} } \right) +  \parallel v \parallel _1.$$

Subject to

$$\mathop \sum \limits_{j = 1}^{n} S_{ij} v_{j} = 0,\,\forall \,\, metabolite \,i,$$

$$0 \le v_{j} \le x_{j} , \,\forall \,\,irrevesible\, reaction \,j,$$

$$- x_{j} \le v_{j} \le x_{j} , \,\forall \,\, revesible\, reaction \,j,$$

where, $$\emptyset \left( u \right) = \left\{ {\begin{array}{*{20}c} {u^{2} , \left| u \right| \le 1} \\ {2\left| u \right| - 1, \left| u \right| > 1.} \\ \end{array} } \right.$$

### Analysis of model predictions for consistency with transcriptome data

The concurrence of the flux predictions and transcriptome data was assessed on both qualitative and quantitative bases. The predicted fluxes were compared with gene expression in developing storage roots of cassava, developing storage roots of high-yield cassava cultivars (KU50 and Arg7), and developing storage roots of the KU50 cassava cultivar. The reactions with zero- and non-zero fluxes were firstly contrasted based on the absence and presence of enzymatic gene expression, respectively. The activity of reactions linked to multiple enzymatic genes was considered depending on the highly expressed genes in the set relative to the thresholds: 25^th^ (low-rank) and 75^th^ (high-rank) percentiles of enzymatic gene expression in the rMeCBM model. The active reactions, predicted non-zero fluxes, were compared with their responsible gene expression, and the consistency was evaluated using a confusion matrix with the following indices: accuracy (ACC), sensitivity (SEN), specificity (SPC), negative predictive value (NPV), and positive predictive value (PPV).

In addition, the sum of square difference (*SSD*; Eq. 4) was employed to measure the distance between the levels of predicted fluxes and responsible gene expression. Percentile ranks of both entities were used to determine the *SSD* as follows:

4 $$SSD_{algorithm, threshold} = \left( {v_{j} - z_{j} } \right)^{2} ,$$

where $v_{j}$ and $z_{j}$ are percentile ranks of the predicted flux and the associated gene expression level of the *j*^th^ reaction, respectively.

### Plausibility analysis

The rMeCBMx-HPCOF model was validated similarly to rMeCBM^16^. The model was employed to simulate the growth of storage roots of cultivar CMC9, to ensure specificity of simulation to the modeled conditions. Besides, the sensitivity of the model was analyzed to support its performance. Simulation of the biomass growth rate was performed under a varying range of *S*_*GAM*_ (stoichiometric coefficients of energy for root growth), for each of which the deviation from the best fit model was determined (Eq. 5*.*)^16^.

5 $$\varepsilon \left( \% \right) = \frac{{v_{p} - v_{o} }}{{v_{o} }} \times 100,$$

where $\varepsilon$ is the percent error of model simulation ($v_{p}$) relative to the measured cassava storage root growth rate $(v_{o}$).

### Model verification by multi-omics data

The predictions of the rMeCBMx were validated with multi-omics data of cassava storage roots, consisting of seven transcriptome data (Wang et al.^32^; Supplementary Table S1), five proteome data (Sheffield et al.^37^, Li et al.^38^, Owiti et al.^39^, Vanderschuren et al.^40^, and Naconsie et al.^41^; Supplementary Table S2), and 20 metabolome data (Drapal et al.^42^ and Obata et al.^43^; Supplementary Table S3). Correspondence of the predicted active and inactive reaction fluxes to the gene expression, proteins and metabolic compounds in cassava storage roots was examined. For the transcriptome data, gene expression was measured from three cassava cultivars at different ages using the Illumina sequencing technology. It should be noted that only genes with expression levels above the 50^th^ percentile rank were used. For the proteome data, existing proteins in developing storage roots were measured from five cassava cultivars through 2D SDS-PAGE and high-resolution liquid chromatography mass spectroscopy (LC-MS). The proteins were BLASTp searched against cassava genome v.6^44^ using identity percentage ≥ 60, coverage percentage ≥ 80 and e-value ≤ 10^−10^. For the metabolome data, metabolic compounds in storage roots were retrieved from 20 studied conditions, including fourteen cassava cultivars planted in different systems. The analyses were based on gas chromatography-mass spectrometry (GC-MS) and LC-MS.

## Results and discussion

### Discrepancies between rMeCBM prediction and metabolic gene expression in cassava storage roots

To study carbon utilization in cassava storage roots, the rMeCBM model is compartmentalized into cytosol, mitochondria, and plastid and covers 468 reactions associated with 393 metabolites, 181 enzymes, and 762 genes. The model was employed to study the partitioning of assimilated carbon in low (HN) and high-yield (KU50) cassava cultivars to determine the metabolic basis for their distinct root biomass. Simulation results obtained for KU50 were comparable to those for CMC-9, a high-yield cultivar from an independent study used for the validation, confirming its reliability, along with the sensitivity analysis of the predictions^16^. However, direct verification of the metabolic fluxes proved elusive, and there remains a knowledge gap between the simulated yield phenotypes and the true metabolic flux distribution in carbon metabolism.

**Figure 2:**
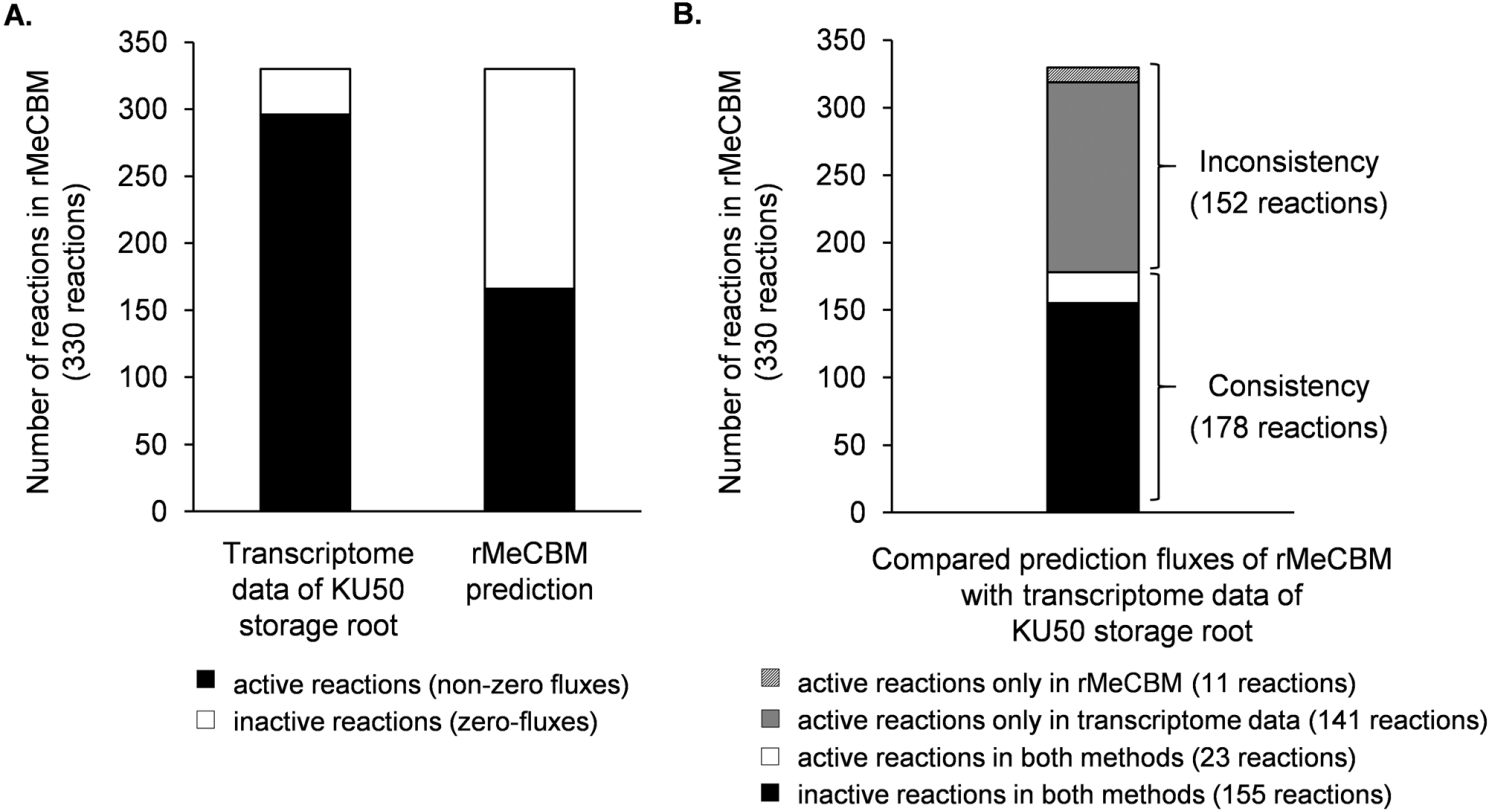
Comparison of 330 biochemical reactions from the rMeCBM model to transcriptome data of developing storage roots of cultivar KU50 at five months old under field conditions, from Wang et al.^32^. (**A**) The number of active and inactive reactions based on transcriptome data of KU50 storage roots and flux prediction from the rMeCBM model. (**B**) Comparison of reactions inferred from transcriptome data and flux prediction (see more details in Supplementary Data S1).

Gene expression is one simple indication of active reactions in the metabolism under a particular condition because metabolic reactions cannot achieve their functions unless related enzymatic genes are transcribed. By assuming that all enzymes functioned independently in the metabolism and were able to modulate each metabolic reaction individually, the active reactions were inferred from at least one expressed enzymatic gene responsible for the reaction. The reactions were considered active if the responsible gene expression was greater than the 50^th^ percentile of overall gene expression in the model. The rMeCBM model was examined with the genome-wide gene expression data of KU50 storage roots at 150 DAP^32^ (Fig. 2, Supplementary Fig. S1 and Supplementary Data 1). The results suggested that the metabolic pathway was partially active under the condition, denoted by 166 non-zero fluxes and 296 gene-expression guided active reactions from a total of 330 biochemical reactions in the rMeCBM model (Fig. 2A). Comparing the metabolic pathway activity based on active-inactive reactions, the model showed 178 predicted flux reactions, 155 non-zero fluxes (active) and 23 zero fluxes (inactive), corresponding to the transcriptome data (black arrows in Supplementary Fig. S1). Despite, almost half of the predictions, 152 of 330 reactions, did not well agree with the transcriptome data. Supplementary Fig. S1 highlights the mis-predicted reactions in the simplified pathway scheme, with blue and red arrows depicting false negative (zero-flux prediction in transcriptome-active reactions) and false positive (non-zero flux for transcriptome-inactive reactions) predictions, respectively. These reactions are related to respiration, amino acid biosynthesis, and the pentose-phosphate pathway, which contains highly branched reactions. About 141 active reactions, by expression data, were predicted to carry zero fluxes in rMeCBM (blue arrows in Supplementary Fig. S1), whereas 11 inactive reactions by expression data carried fluxes in the simulation (red arrows in Supplementary Fig. S1, Fig. 2B). The rMeCBM model could not capture the cytosolic conversion of sugar phosphate to pyruvate in the respiration pathway for ATP production (marked as I. in Supplementary Fig. S1) and contradictorily predicted flux through serine-pyruvate transaminase (EC 2.6.1.51; R00585) reaction in the complex alanine biosynthesis pathway (marked as II. in Supplementary Fig. S1). These pathways are essential for energy production and biomass synthesis in cells. Studies in other plants have shown that glycolytic fluxes in cytosol were used to generate triose-phosphate and precursors for the TCA cycle^14,19^.

### Transcriptome-integrated metabolic models of carbon assimilation in developing storage roots of cassava

**Figure 3:**
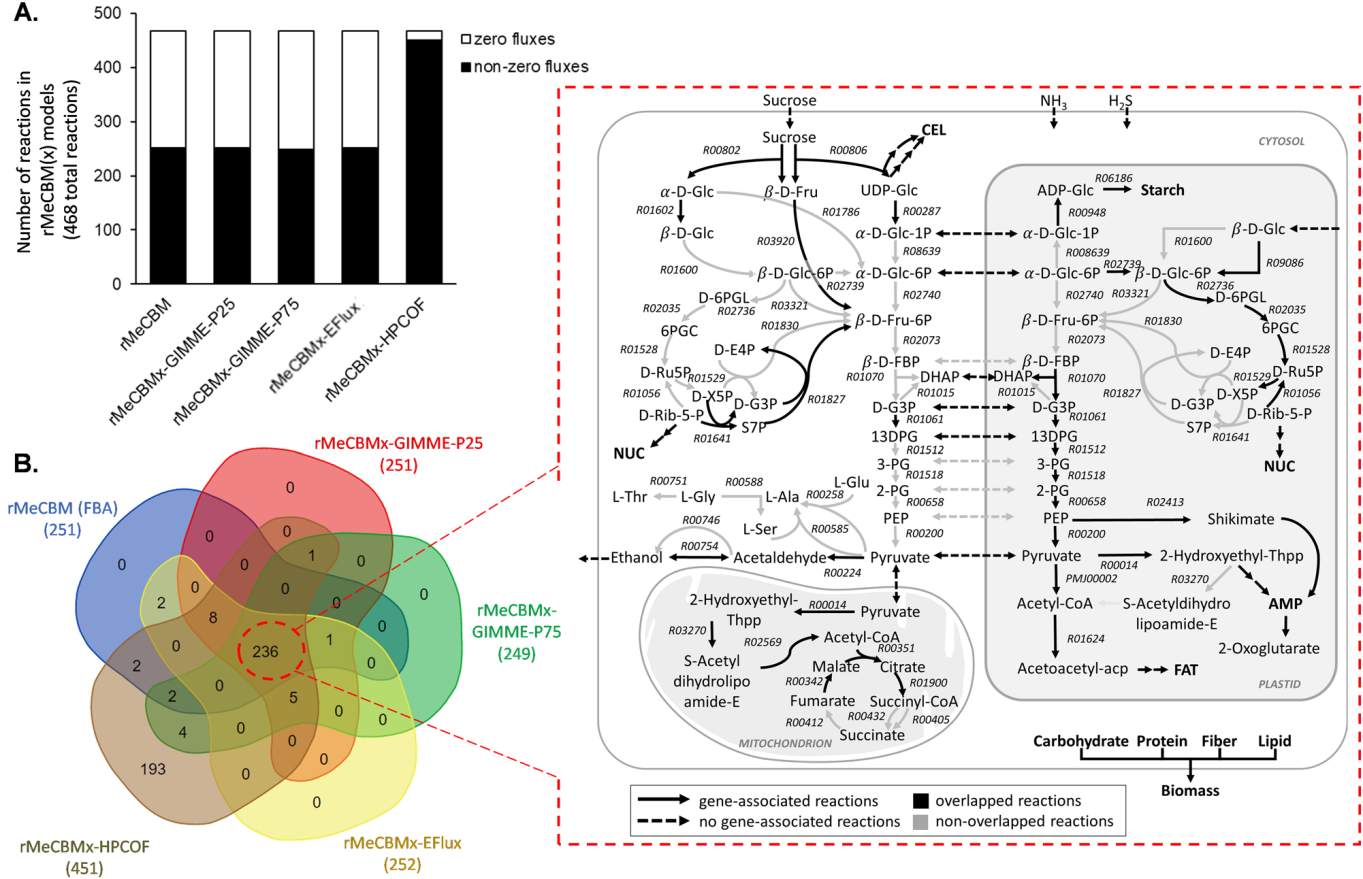
Simulation of the transcriptome-integrated CBM models (rMeCBMx) constructed using GIMME, E-Flux and HPCOF algorithms, (**A**) the number of non-zero (active) and zero (inactive) flux reactions from rMeCBM, rMeCBMx-GIMME-P25, rMeCBMx-GIMME-P75, rMeCBMx-EFlux, and rMeCBMx-HPCOF and (**B**) commonly predicted active reactions from all models, 236 of 468 total reactions denoted in the overlapped region of the Venn diagram and depicted in the schematic pathway as black solid arrows, postulated to be core metabolic reactions basically driving carbon assimilation in developing storage roots of cassava. Gray arrows denote trivial reactions obtained from some model predictions. The solid and dotted arrows represent genes associated with the reactions.

To improve metabolic flux prediction of carbon assimilation in cassava storage roots, gene expression data were incorporated to confine the optimization of the basic constraint-based model (rMeCBM), hereafter referred to as rMeCBMx. The storage root transcriptome of 3-month-old plants^31^ was employed to represent the gene expression landscape in the root. The dataset was one of the only few RNA-Seq measurements with multiple replications that investigated in a similar stage of storage root development to the previous rMeCBM study. The integrative model was constructed based on three different conceptual methods, GIMME, E-Flux, and HPCOF, to ensure optimal methodological setting. The models were simulated to fit the specific growth rate of the KU50 cultivar (Supplementary Table S4). GIMME uses the transcriptome data to guide the set of active reactions by assuming that reactions are supposed to be active once the expression of related genes is greater than a threshold and minimizes flux through inactive reactions^23^. In our analysis, the active reactions were determined if the expression of related genes were above the 25^th^ and 75^th^ percentiles of overall metabolic gene expression in rMeCBM, representing the maximal and minimal set of active reactions, respectively (Fig. 3A, Supplementary Figs. S2, S3, Supplementary Table S5). The rMeCBMx-GIMME-P25 (25^th^ percentile) model predicted 251 non-zero flux (active) and 217 zero-flux reactions, including two reactions with inferior gene expression recovered from predefined inactive reactions during optimization (Supplementary Figs. S2, S3A, Supplementary Table S5), while 249 non-zero flux (active) and 219 zero-flux reactions, including 62 recalled from the predefined inactive reactions (Supplementary Figs. S2, S3B, Supplementary Table S5) were predicted at the 75^th^percentile threshold (rMeCBMx-GIMME-P75). These models showed improved flux prediction relative to rMeCBM, owing to the metabolic gene expression information. They predicted the use of pyruvate-glutamate transaminase (EC 2.6.1.2; R00258) to synthesize alanine instead of serine-pyruvate transaminase (EC 2.6.1.51; R00585). The rMeCBMx-GIMME-P25 and rMeCBMx-GIMME-P75 showed similar predictions but not identical. The carbon precursors imported from the cytosol for utilization in the plastid were different. rMeCBMx-GIMME-P75 preferred beta-D-Fructose 1,6-bisphosphate ($\beta$-D-FBP) in the plastid similar to the rMeCBM model, while rMeCBMx-GIMME-P25 imported alpha-d-glucose-6-phosphate ($\alpha$-d-Glc-6P) for the respiration pathway and biosynthesis of other biomass components. Hill and Smith^45^ reported that $\alpha$-d-Glc-6P is imported as the substrate for starch synthesis in the plastids of developing pea embryos^45^. Additionally, the models were different in the use of the bypass reaction R01830 in non-oxidative PPP for the conversion of d-erythose-4-phosphate (d-E4P) and d-xylulose-5-phosphate (d-X5P) to d-glyceraldehyde-3-phosphate (d-G3P) and beta-d-fructose-6-phostphate ($\beta$-d-Fru-6P) by transketolase (EC 2.2.1.1). rMeCBMx-GIMME-P25 predicted R01830 in the plastid, whereas rMeCBMx-GIMME-P75 predicted it in the cytosol. The activity of PPP in the cytosol and plastid is complicated and varies in several plants. Cellular fractionation studies with leaves and roots of maize, pea, and spinach have provided evidence that the non-oxidative reactions are confined to the plastids^46^. Debnam and Emes^47^ found non-oxidative enzymes in cytosolic and the plastidic compartments in tobacco roots and leaves^47^. Moreover, rMeCBMx-GIMME-P75 predicted higher fluxes of non-cyclic TCA than rMeCBMx-GIMME-P25. The results demonstrated the GIMME integrative model could improve flux prediction of rMeCBM; however, the prediction was strongly dependent on the set threshold. The flux variability analysis (FVA) showed the variation in fluxes predicted at different threshold settings by the GIMME models was mostly from substitutable reactions, defined according to Hay and Schwender^14^, indicating viable alternative solutions for the prediction (Supplementary Fig. S2). The FVA also supported the essentiality of the recovered low gene expression reactions. These reactions are related to amino acid and fatty acid biosynthesis pathways, which are essential for storage root growth. Inference of active and inactive reactions based on a specified threshold, a key component of GIMME, introduces arbitrariness into the prediction.

E-Flux was introduced here as an integration method that is independent of arbitrary thresholds. It incorporates gene expression data to approximate maximal enzymatic activity for biochemical conversions, implemented as an upper bound of viable flux prediction^25^. The rMeCBMx-EFlux model predicted 252 non-zero flux reactions and 216 zero-flux reactions (Fig. 3A, Supplementary Fig. S4). While E-Flux had an advantage over GIMME, particularly regarding its ability to capture reactions with low gene expression and the absence of arbitrary thresholds, predicted fluxes were similar to rMeCBM with subtle improvements. There were, however, differences in the carbon precursors imported from the cytosol for utilization in the plastid. rMeCBMx-EFlux transported $\alpha$-d-Glc-6P into plastid for the respiration pathway and biosynthesis of other biomass components instead of $\beta$-d-FBP.

In a similar manner to E-Flux, HPCOF constrains a model directly to gene expression levels but predicts fluxes through robust global optimization^28^. The HPCOF model predicts a large number of non-zero fluxes of varied magnitudes because it optimizes metabolic fluxes based on gene expression instead of an attempt to eliminate inactive reactions. The rMeCBMx-HPCOF model predicted 451 non-zero flux reactions and 17 zero-flux reactions (Fig. 3A and Supplementary Fig. S5). It was the only model that could capture the conversion of sugar phosphate to pyruvate through glycolysis and the pentose phosphate pathway in both the cytosol and plastid, with the full cycle of mitochondrial TCA. Among the methods, HPCOF captured the highest number of active metabolic reactions; however, its predictions might have been overestimated due to high post-transcriptional regulatory effects. Despite their uniqueness, the models did show some overlaps, particularly with respect to the main reactions related to the biosynthesis of starch, amino acids, fatty acids, and plastidic glycolysis (Fig. 3B).

### Biological inference of the transcriptome-constrained metabolic model of cassava storage roots

The four transcriptome-constrained metabolic models, rMeCBMx-GIMME-25, rMeCBMx-GIMME-75, rMeCBMx-EFlux, and rMeCBMx-HPCOF, were assessed for their ability to better predict carbon assimilation in cassava storage roots. Metabolic flux prediction of each model was contrasted with gene expression data collected from various transcriptomic studies to represent metabolic processes in cassava storage roots. First, the predictions were compared with 472 commonly expressed genes linked to metabolism in developing storage roots of cassava (scenario 1) from eight datasets^31,32^. Next, the comparison was performed using six datasets^32^ containing 545 commonly expressed genes associated with metabolic processes in developing storage roots of high-yield cassava cultivars KU50 and Arg7 (scenario 2). Lastly, the predictions were compared with 589 genes commonly expressed in developing storage roots of KU50 cultivar (scenario 3) across three datasets^32^ (Supplementary Table S6). The difference in the number of expressed genes in each scenario may explain the specificity of traits. GO functional analysis showed a majority of enriched GO terms in the three groups of transcriptome datasets (scenarios) were quite similar, particularly 175 enriched GO terms, and were mostly related to carbohydrate and nucleotide metabolism (Supplementary Table S7, Supplementary Fig. S6A–C), which play major roles in starch accumulation in developing cassava storage roots. Moreover, eight GO terms mainly associated with steroid metabolism were found in developing storage roots of the high-yield cultivar KU50. Steroids exert a wide range of biological activities, which are essential for plant growth, reproduction, and responses to various abiotic and biotic stress^48^. A total of 18 enriched GO terms that are related to homeostasis were found only in developing storage roots of the KU50 cultivar. In addition, the enriched GO terms of each scenario were explored at low (25^th^ percentile) and high (75^th^ percentile) thresholds. The functional modules were slightly different at both cutoffs (Supplementary Fig. S6B,C). Glutamine metabolism, steroid metabolism, and glycopeptides alpha-*N*-acetylgalactosaminidase activity were only found at the low-threshold in developing cassava storage roots of the high-yield cultivar (Supplementary Fig. S6B). On the other hand, active UDP-glycosyltransferase activity and carbohydrate derivative biosynthetic process were mainly found in highly expressed genes (Supplementary Fig. S6C).

**Figure 4:**
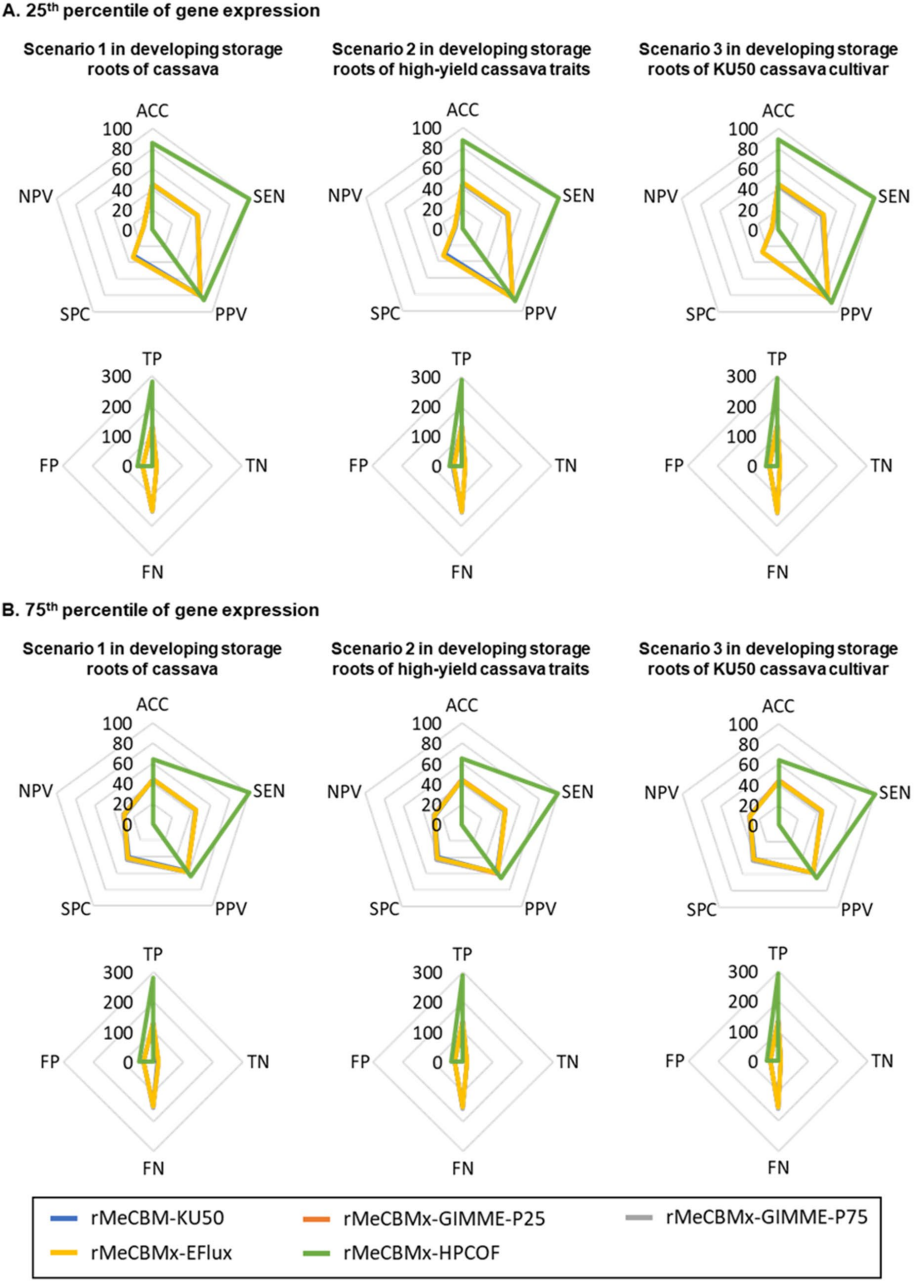
The qualitative analysis of model performance based on three algorithms at varied thresholds of expressed genes from transcriptome data (see more details in Supplementary Fig. S7).

**Figure 5:**
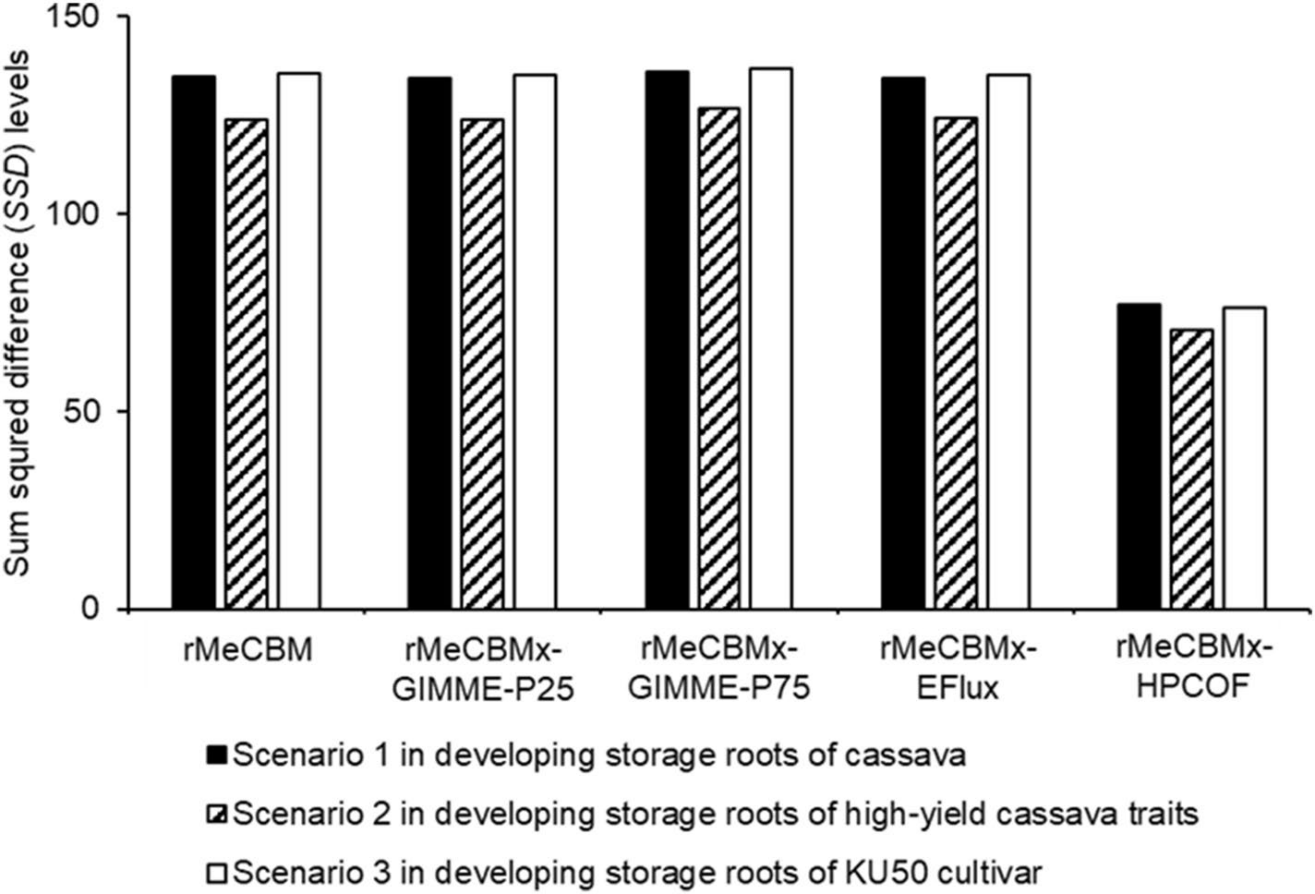
The sum of squared difference (*SSD*) between ranked predicted fluxes from each algorithm and ranked gene expression levels in specific conditions of cassava storage roots. The lower *SSD* indicates the higher consistency between flux prediction and gene expression levels.

The consistency of the predicted fluxes with the expression of enzymatic genes responsible for the reactions was determined based on moderately-to-highly expressed genes. Primarily, the predicted fluxes were employed to denote active reactions involved in carbon assimilation in developing storage roots. The predicted active reactions that carry non-zero fluxes were assessed by the expression of related enzymatic genes from transcriptomic studies. The performance of the models in terms of representing carbon assimilation in cassava storage roots was evaluated through multiple indexes of confusion matrix, including accuracy (ACC), sensitivity (SEN), specificity (SPC), negative predictive value (NPV), and positive predictive value (PPV). The rMeCBMx-HPCOF model outperformed the other models and accurately predicted flux through active reactions (~ 80%) in developing storage roots with high sensitivity (~ 85%). Unlike others, rMeCBMx-HPCOF was able to capture particular functions in developing storage roots of the KU50 cultivar, such as the conversion of beta-d-Glucose ($\beta$-d-Glc) to beta-d-Glucose 6-phosphate ($\beta$-d-Glc-6P) (denoted as R01600), synthesis of d-Ribose 5-phosphate (d-Rib-5-P) using 6-Phospho-d-gluconate (d-6PGL) as a substrate in the oxidative pentose phosphate pathway in the cytosol, and conversion of sugar phosphate to pyruvate in the cytosol. rMeCBMx-HPCOF showed high positive predictions (active reactions) with few negative predictions (inactive reactions). The predictive performance of the models was comparable at high gene expression (> 75^th^ percentile, Fig. 4). Nonetheless, rMeCBMx-HPCOF showed superior predictive performance overall. Furthermore, we quantitatively examined the consistency of predicted flux values and the levels of gene expression based on the sum of squared differences (SSD). Our results showed that rMeCBMx-HPCOF had the lowest SSD, indicating high prediction confidence (Fig. 5). Accordingly, rMeCBMx-HPCOF was employed for further investigation of metabolic phenotypes in developing cassava storage roots.

### Plausibility of rMeCBMx-HPCOF

To show that rMeCBMx-HPCOF better simulates physiological growth of developing storage roots of cassava and provides a more insightful prediction of metabolic flux than the previous FBA model (rMeCBM), rMeCBMx-HPCOF was validated similarly to the original model, employing the KU50 and CMC-9 cultivars, and its sensitivity to the *S*_*GAM*_ was determined. rMeCBMx-HPCOF perfectly simulated the specific growth rate of the KU50 cultivar, 0.0090 day^−1^, with *S*_*GAM*_ equal to 19.7 mmol_ATP_ gDW^−1^ (Supplementary Table S4). The optimal *S*_*GAM*_ was higher than for the previous model, but was still in the 5–42 mmol_ATP_ gDW^−1^ range reported for other metabolic models of plants, including Arabidopsis^11^, rice^49^, maize^15^, barley^50^, and rapeseed^51^. The optimal *S*_*GAM*_ was seemingly overestimated than would be expected in reality when compared to measured ATP levels in potato tubers (0.36–0.55 μmol/gDW)^52^. To acquire better ATP predictions, the model needs to take into account full costs of ATP usage, e.g. for cell maintenance and metabolite transportation^19^. This issue is worth revisiting when essential data such as energy-linked transporters for nutrient uptake and intercellular transportation become available for cassava.

The simulation was also comparable to the measured growth rate of CMC-9^53^, and the predictive performance, to the basic rMeCBM model (Supplementary Fig. S8). Moreover, the sensitivity of rMeCBMx-HPCOF to *S*_*GAM*_, a key variable indicating the use of ATP for storage root growth, was investigated against the predicted growth rate of cassava storage roots at up to 20% deviation from the optimal growth rate. Results showed rMeCBMx-HPCOF was slightly more robust than rMeCBM at 15.75–23.64 mmol_ATP_ gDW^−1^_storage roots_ and 7.84–12.74 mmol_ATP_ gDW^−1^_storage roots_, respectively (Supplementary Fig. S9).

**Figure 6:**
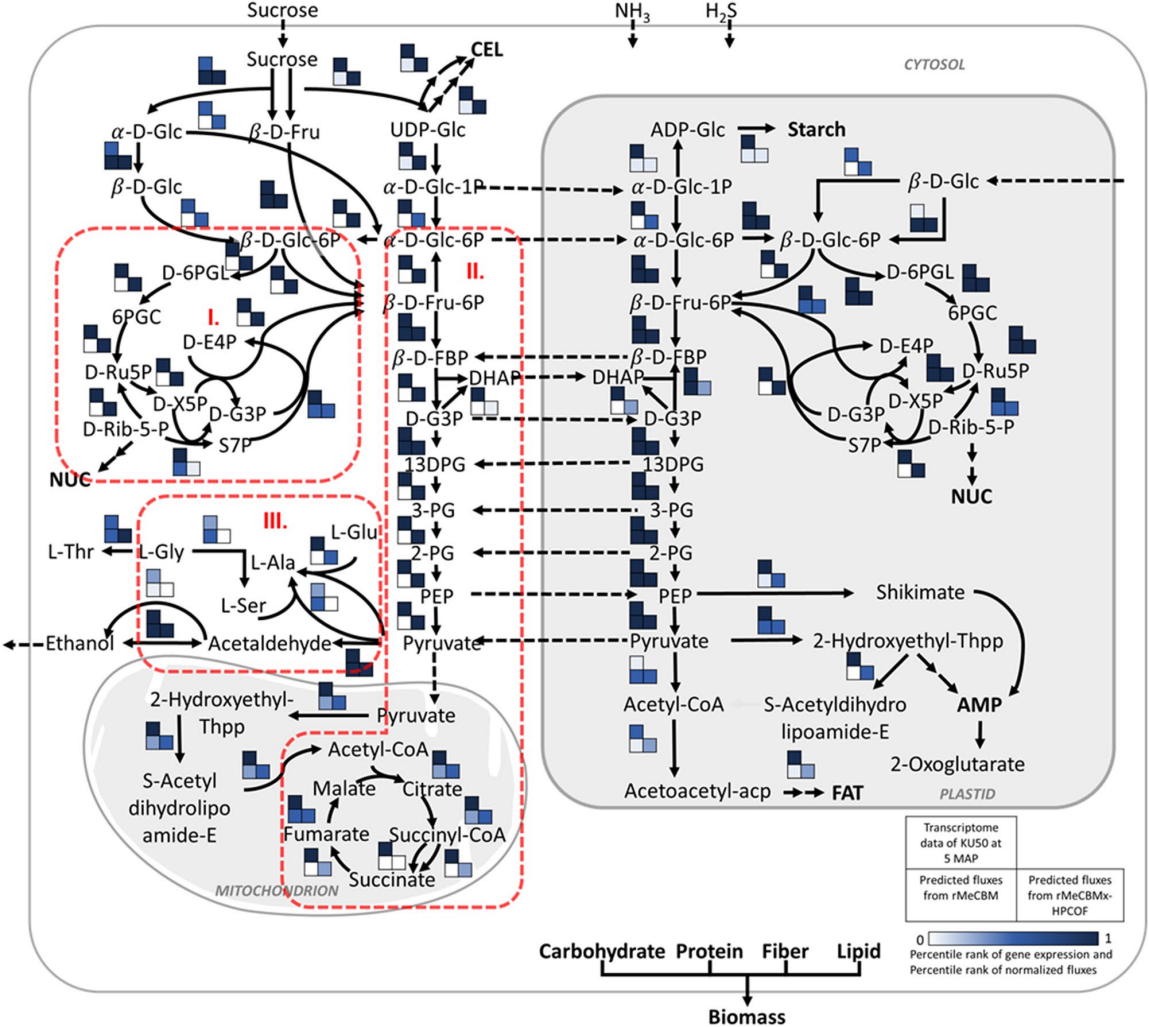
The comparison of ranked gene expression levels (top cell) with ranked fluxes from rMeCBM (left bottom cell) and rMeCBMx-HPCOF (right bottom cell) as shown in the heatmap; the color represents relative levels of gene expression and fluxes with a score of 0–1. The improvement of rMeCBMx-HPCOF prediction is linked to (I) carbon substrates supply via pentose phosphate pathway, (II) TCA cycle and glycolysis, (III) carbon precursors for alanine biosynthesis.

According to this analysis, we assured that rMeCBMx-HPCOF was able to simulate the physiological growth of cassava storage roots similarly to rMeCBM but provided more precise flux predictions that corresponded to enzymatic gene expression in developing storage roots. Three main areas of carbon metabolism were, in particular, improved by rMeCBMx-HPCOF, namely (I) carbon substrates supply via the pentose phosphate pathway, (II) TCA cycle and glycolysis in the respiration pathway, and (III) carbon precursors for alanine biosynthesis (Fig. 6).

#### Carbon substrates supply via pentose phosphate pathway

The pentose phosphate pathway (PPP), a maintenance pathway, is crucial for the biosynthesis of amino acids and nucleotides and is a major source of NADPH, which is required against oxidative stress and for the synthesis of fatty acids. The PPP is composed of oxidative and non-oxidative phases located in both cytosol and plastid. The PPP is not a simple linear pathway since several carbon atoms are recycled back into glycolysis. Predicting the metabolic activity in PPP by a computational approach is challenging^54^. The prediction of oxidative PPP by FBA differed with the 13C-MFA (metabolic flux analysis) experiment, in a study of fluxes in central metabolism in heterotrophic Arabidopsis cells under stress conditions^18^. The rMeCBMx-HPCOF model predicted the oxidative PPP (OPPP) pathway, the conversion of $\beta$-d-Glc-6P into d-Ru5P through the sequential actions of glucose-6-phosphate dehydrogenase (G6PDH) and 6-phosphogluconate dehydrogenase (6PGDH), with lactonase catalyzing the hydrolysis of its d-6PGL product, in both the cytosol and plastid, unlike rMeCBM, which only captured the oxidative PPP in the plastid. The results were corroborated by Krook et al*.*^55^, who reported the existence of oxidative pentose phosphate pathway-mediated sugar converting cycles in both the cytosol and plastid, based on the redistribution of labeled hexoses in carrot cells grown in batch culture on [13C]-labeled glucose or fructose^55^. In higher plants, the OPPP provides high amounts of NADPH required for reductive biosynthesis and protection against oxidative damage in the cytosol and plastids, providing key intermediates, C-5 sugar phosphates (e.g., d-Rib-5-P and d-E4P) for the shikimate pathway to synthesize amino acids, which is an essential process during storage root formation^56,57^. The cytosolic G6PDH gene is crucial for the supply of NADPH required for plant defense responses to pathogenic infection in tobacco^58^, salt stress in Arabidopsis roots^59^, and drought stress in soybean roots^57^. Deletion of the G6PDH gene results in the overproduction of reaction oxygen species (ROS), reductions in root elongation and germination rate of plants under stress, and a decrease in plant productivity^57,59^. Overexpression of the G6PDH gene induced drought tolerance in transgenic tobacco^58^ and increased the length of roots during early development in rice^60^. Regulation of the G6PDH enzyme in the chloroplast of photosynthetic tissues is governed by the ferredoxin/thioredoxin system under darkness, while high sugar levels in cytosol of non-photosynthetic tissue enhance the transcription of the G6PDH gene^61^. The activated G6PDH predicted in developing storage roots of cassava may reflect the metabolism of high sugar levels to support starch biosynthesis and accumulation in the root parenchyma^61^.

#### TCA cycle and glycolysis

The TCA and glycolysis pathways are related to ATP production in cells. The main function of glycolysis is to oxidize hexoses to provide ATP, pyruvate, and precursors for anabolism. Glycolysis is a crucial process in plant because it is the predominant pathway that “fuels” plant respiration and also provides sugar phosphates and carbon substrates for the biosynthesis of numerous compounds such as amino acids, starch, and cellulose^62^. This process occurs in both cytosol and plastids of plant cells^62,63^. The rMeCBM model could not capture the glycolysis process in the cytosol as pyruvate was generated only in the plastid and then exported into the cytosol, whereas rMeCBMx-HPCOF could. This result is relevant to the transcriptome analysis of cassava storage roots from Yang et al.^64^, who proposed enolase, l-lactate dehydrogenase and aldehyde dehydrogenase for glycolysis/gluconeogenesis as rate-limiting enzymes that are essential for starch biosynthesis in storage roots. Voll et al.^65^ reported that antisense inhibition of enolase, which catalyze the conversion of 2-phosphoglycerate (2-PG) and phosphoenolpyruvate (PEP), strongly limits the levels of PEP and amino acids in transgenic tobacco leaves because PEP is a precursor for aromatic amino acids in the shikimate biosynthesis pathway^65^. PEP can be converted by cytosolic pyruvate kinase to pyruvate, which is an important substrate for the TCA cycle. Lack of enzymes in the cytosol might affect the synthesis of buildings blocks for amino acids and other biomolecules of storage roots. Additionally, the HPCOF simulation results showed the complete metabolic process in the mitochondrial TCA cycle that might have resulted from the presence of glycolysis in the cytosol. Jenner et al.^66^ reported that downregulation of the NAD+ dependent malic enzyme (NADME) in the TCA cycle did not affect the TCA cycle activity but significantly increased starch yield in potato tubers^66^, meaning that the regulation of AGPase might depend on the exchange of compounds from central carbon metabolism^67^.

#### Carbon precursors for alanine biosynthesis

The rMeCBM model predicted that l-alanine (l-Ala) was synthesized using l-serine (l-Ser) as a substrate, but it was not consistent with the gene expression as the related genes of serine-pyruvate transaminase (EC 2.6.1.51) showed very low expression levels (~ 0.12 FPKM). On the other hand, rMeCBMx-HPCOF predicted l-Ala biosynthesis using l-glutamate (l-Glu) as a precursor (R00258). Several pathways of l-Ala synthesis have been proposed, the most likely being the formation of l-Ala from l-Glu and pyruvate by transamination through glutamate-pyruvate aminotransferase (GPT; EC 2.6.1.2)^68^. The prediction was relevant to Good et al.^69^, who reported that alanine is a major product of anaerobic metabolism in barley root by pyruvate-glutamate transaminase^69^. GPT, also known as alanine aminotransferase, has been widely studied using genetic engineering to enhance nitrogen use efficiency (NUE) in crop plants^70,71^. l-Alanine acts as an intercellular nitrogen and carbon shuttle and plays a key role in carbon and nitrogen metabolism in plants^72,73^. Overexpression of GPT successfully increased yield in canola, rice^72^, wheat^74^, and sugarcane^75^.

**Figure 7:**
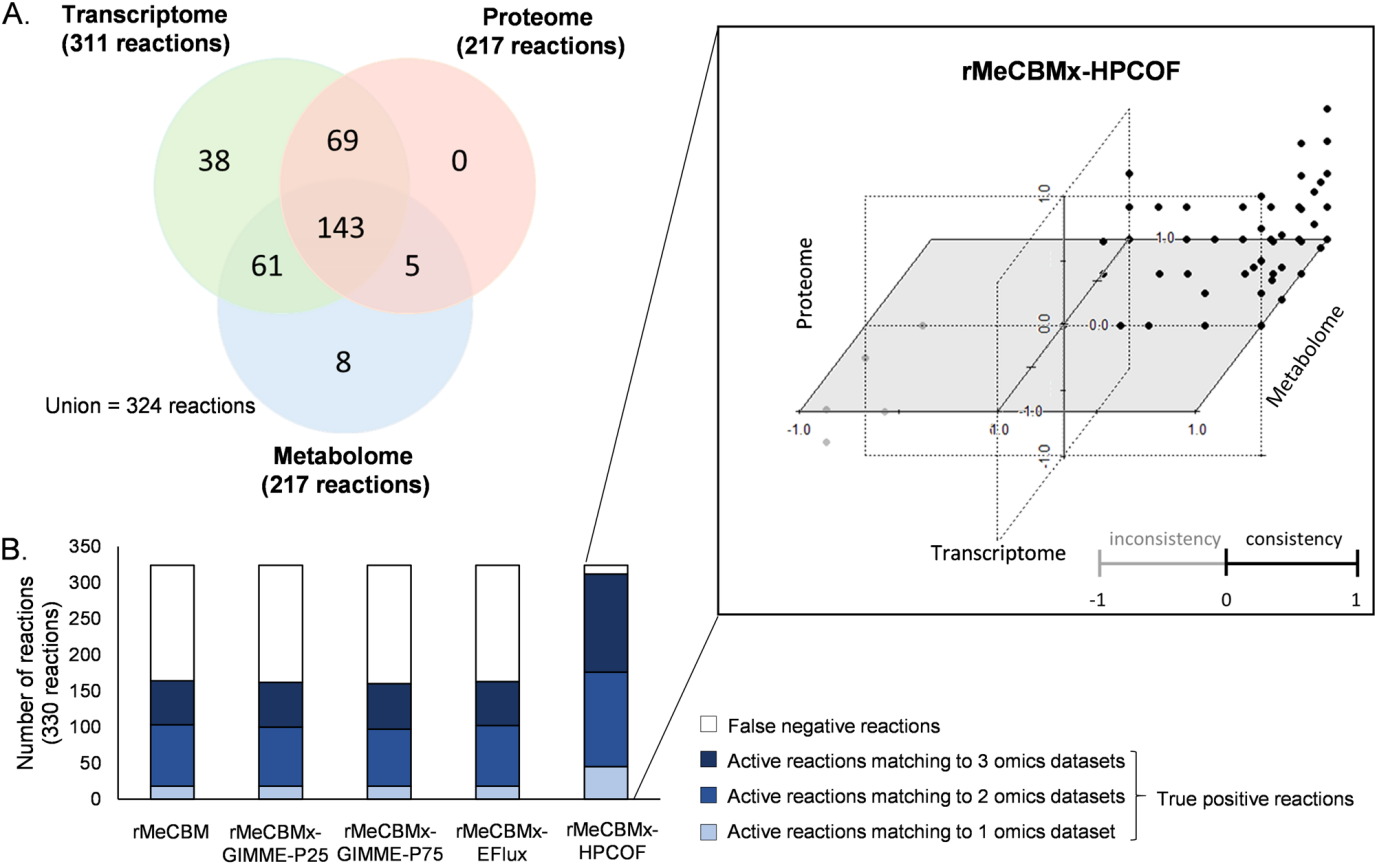
Flux verification by multi-omics data. (**A**) Reactions were indicated to be active based on transcriptome, proteome, and metabolome data. (**B**) Comparison of active reactions from omics data with the flux prediction from each model; rMeCBMx-HPCOF shows the highest consistency of flux prediction with the three omics data, including transcriptome, proteome, and metabolome data, as shown in the graph on the right side.

Moreover, we ensured flux prediction by using multi-omics data to support active flux reactions either by gene expression, protein expression, or availability of the substrate/product metabolites in developing storage roots of cassava. In total, 473 expressed genes from seven transcriptome datasets, 192 expressed proteins from five proteome datasets, and 53 available metabolites from 20 metabolome datasets were used to infer active reactions (Supplementary Fig. S10). From 330 gene-associated reactions in rMeCBMx, predicted flux reactions verified from the transcriptome, proteome, and metabolome data totaled 311, 217, and 217, respectively. In sum, 143 predicted flux reactions were verified from the integrated multi-omics (Fig. 7A). Among them, rMeCBMx-HPCOF could predict 136 reactions, while the other models captured 53–57 reactions, as shown by the dark blue bar in Fig. 7B (see Supplementary Fig. S11 for details of other models).

Our findings indicate that omics constraints are needed to enforce a particular metabolic behavior in carbon metabolism of developing cassava storage roots, and the HPCOF algorithm is the best method in this case to integrate transcriptome data. However, it is of importance to note that rMeCBMx-HPCOF requires further improvements in terms of its ability to predict inactive reactions. Moreover, gene information for transport reactions is still lacking, and gene–protein-reaction associations of cassava storage roots need to be improved.

## Conclusions

Transcriptome-integrated constraint-based metabolic models of cassava storage roots (rMeCBMx) were developed by incorporating gene expression data of cassava storage roots into the genome-scale metabolic model of cassava storage roots (rMeCBM) to reduce infeasible metabolic fluxes. Among three different conceptual algorithms, GIMME, E-Flux, and HPCOF, rMeCBMx-HPCOF showed the highest accuracy to predict carbon fluxes in the metabolism of storage roots and was, in particular, highly consistent with the transcriptome of high-yield cultivars. It improved flux prediction through (1) carbon substrates supply via pentose phosphate pathway, (2) TCA cycle and glycolysis, and (3) carbon precursors for alanine biosynthesis. The flux prediction also was evaluated by proteome and metabolome data of developing cassava storage roots. However, this study only takes advantage of expression data; the inclusion of high-quality physiology and other omics data might increase its predictive power.

## Supplementary Information

Supplementary Dataset.

Supplementary Figures.

Supplementary Tables.
