# Supplementary material for: Transcriptome integrated metabolic modeling of carbon assimilation underlying storage root development in cassava
Source: Sci Rep. 2021 Apr 22;11:8758. doi: 10.1038/s41598-021-88129-3 (PMC8062692; doi:10.1038/s41598-021-88129-3)

Transcriptome integrated metabolic modeling of carbon assimilation underlying storage root development in cassava

Ratchaprapa Kamsen[1]**, Saowalak Kalapanulak**[1,2]**, Porntip Chiewchankaset**[2]**, and Treenut Saithong**[1,2,*]

[1]Bioinformatics and Systems Biology Program, School of Bioresources and Technology and School of Information Technology, King Mongkut's University of Technology Thonburi (Bang Khun Thian), Bangkok, 10150, Thailand.
[2]Center for Agricultural Systems Biology, Systems Biology and Bioinformatics Research Group, Pilot Plant Development and Training Institute, King Mongkut's University of Technology Thonburi (Bang Khun Thian), Bangkok, 10150 Thailand
[*]corresponding author; e-mail: treenut.sai@kmutt.ac.th

Supplementary Fig. S1 Comparison of active reactions in carbon assimilation pathway in storage roots of cassava inferred based on predicted flux distribution and transcriptome data of cultivar KU50 at five months old[1]

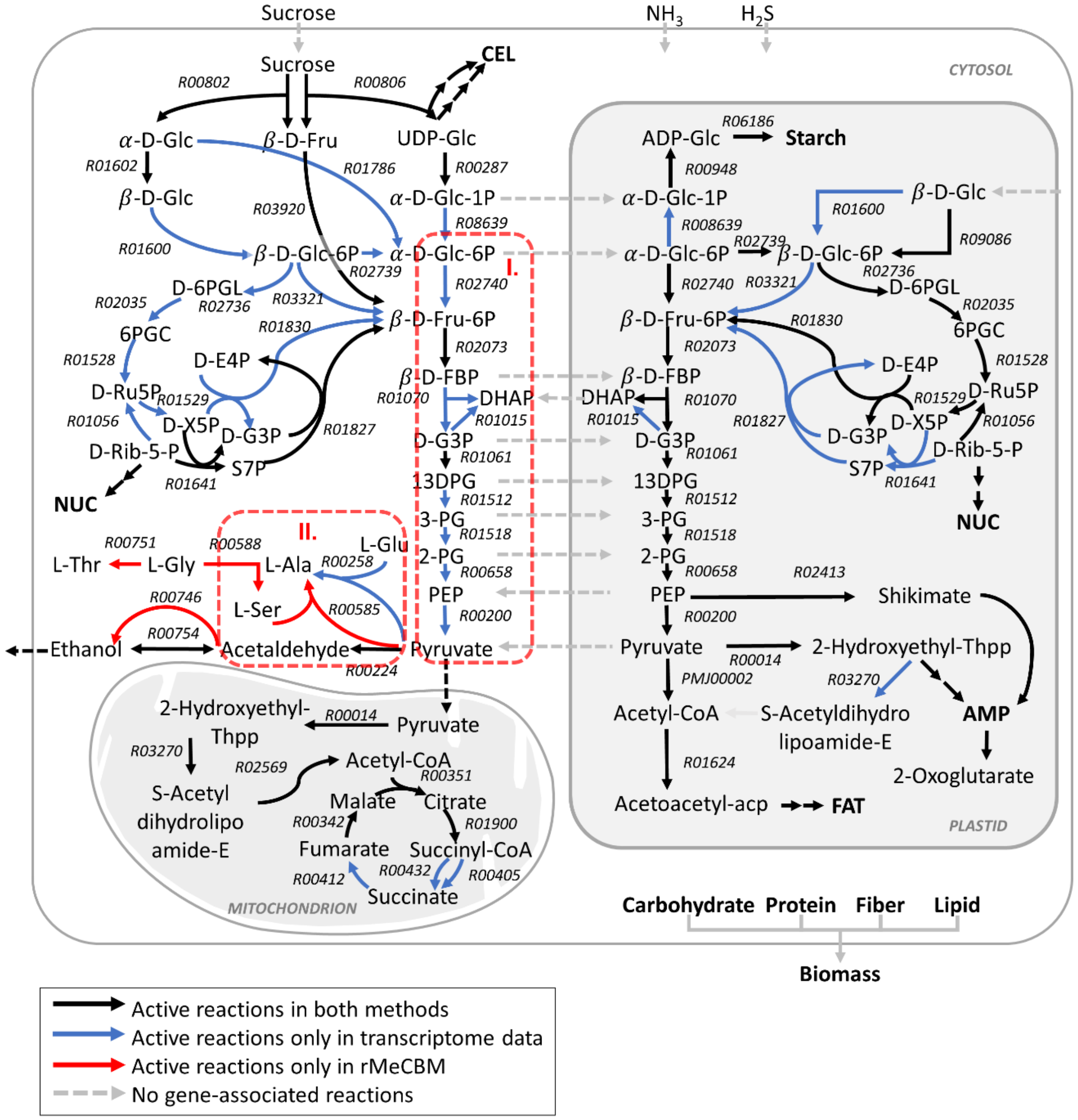

Red and blue arrows represent the reactions that did not well agree with the transcriptome data. The rMeCBM model could not capture the cytosolic conversion of sugar phosphate to pyruvate in the respiration pathway (I.) and the predicted flux through serine-pyruvate transaminase reaction (R00585) in the complex alanine biosynthesis pathway was inconsistency with gene expression (II.).

Supplementary Fig. S2 Flux distribution at varied thresholds and flux variability analysis indicating flux variability types

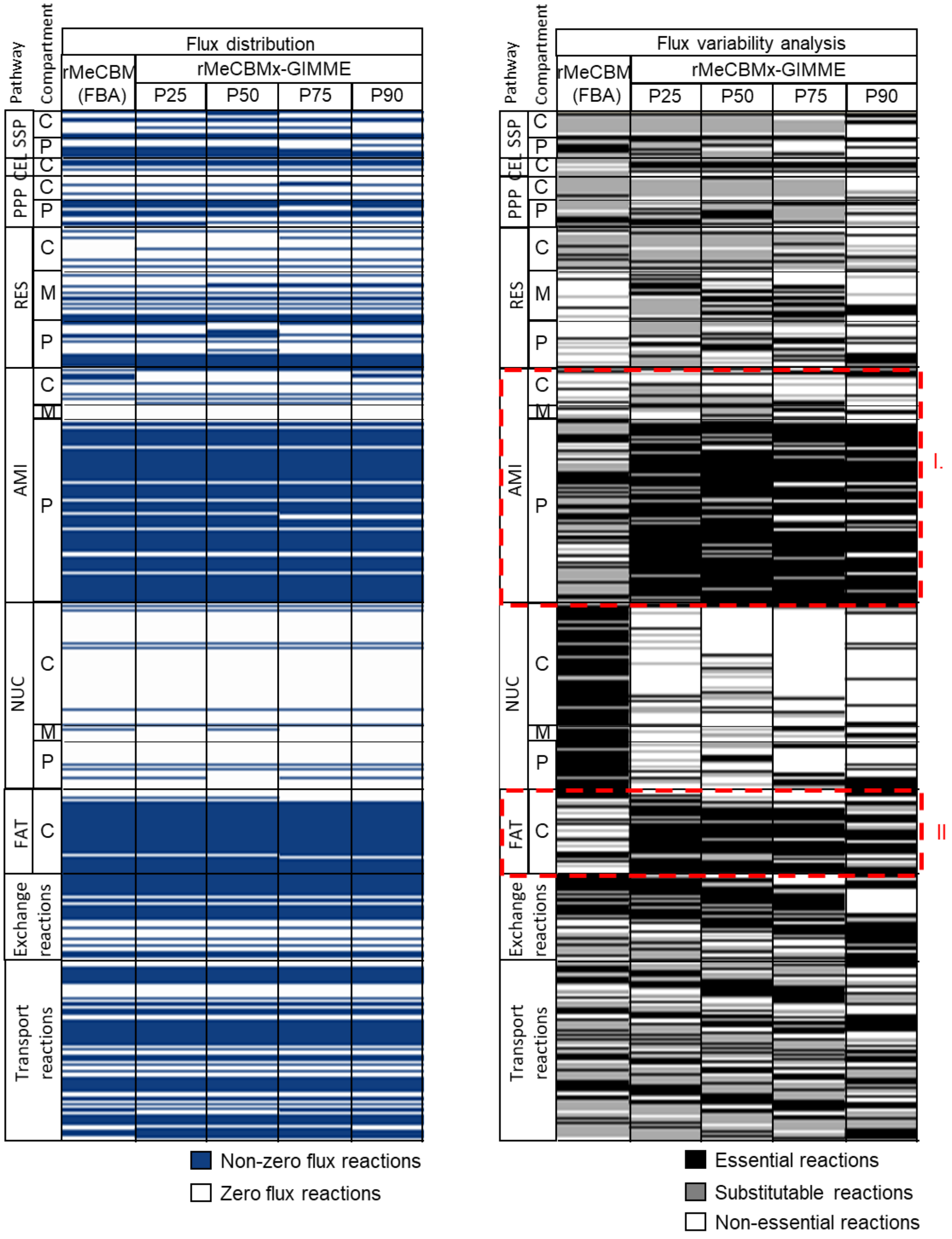

Pathways: starch and sucrose biosynthesis pathway (SSP), respiration pathway (RES), pentose phosphate pathway (PPP), cell wall biosynthesis pathway (CEL), amino acid biosynthesis pathway (AMI), fatty acid biosynthesis pathway (FAT), and nucleotide biosynthesis pathway (NUC).

Compartments: cytosol (c), mitochondria (m), and plastid (p).

GIMME recovered low gene expression reactions. The FVA supported that those recovered reactions are essential reactions (black) related to amino acid (I.) and fatty acid biosynthesis (II.) pathways, with need for storage root growth.

Supplementary Fig. S3 The flux distributions using GIMME algorithm (rMeCBMx-GIMME) at two different thresholds between 25th and 75th percentile of expression levels compared with transcriptome data of cultivar KU50 at five months old

A. rMeCBMx-GIMME-P25

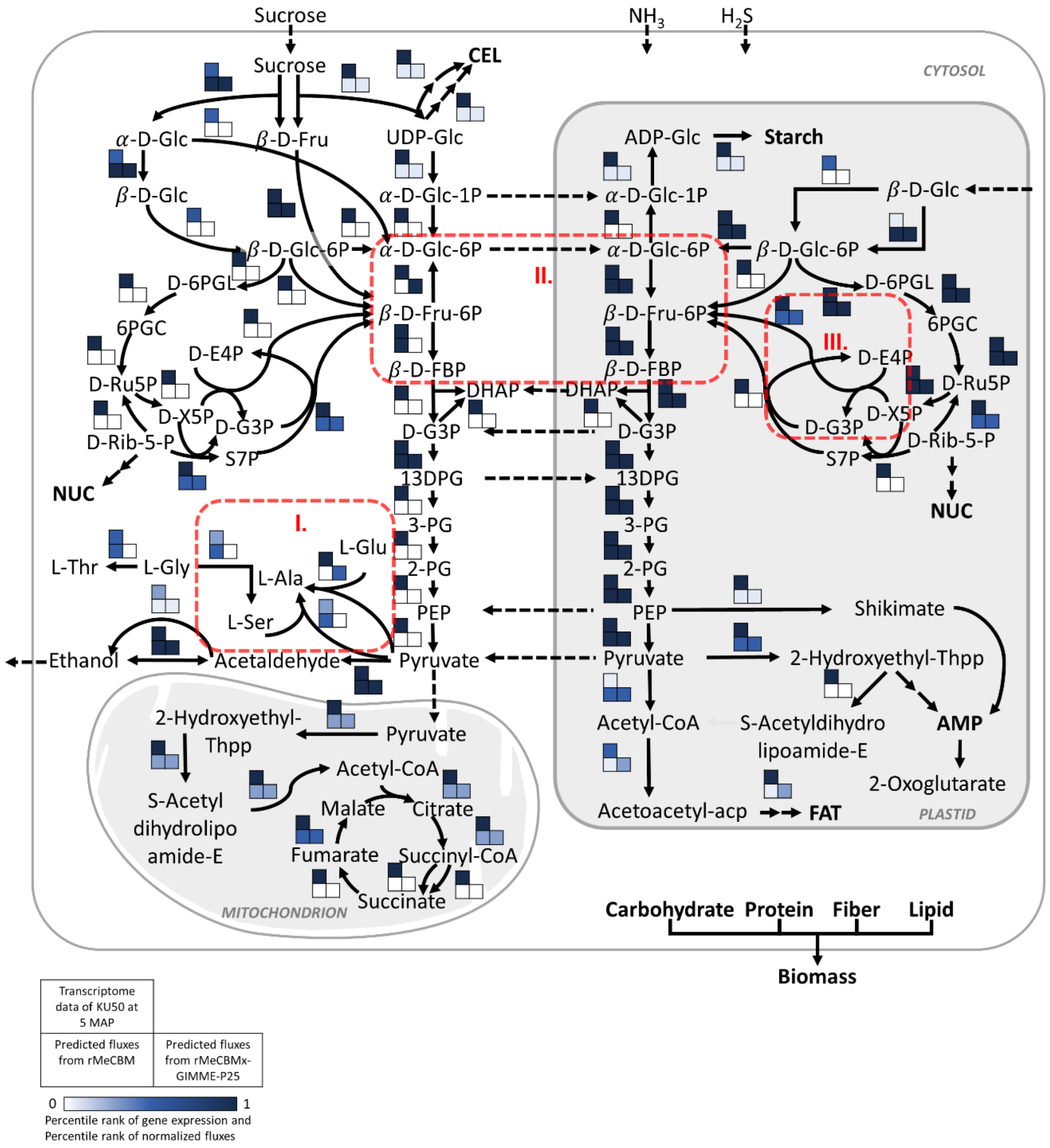

B. rMeCBMx-GIMME-P75

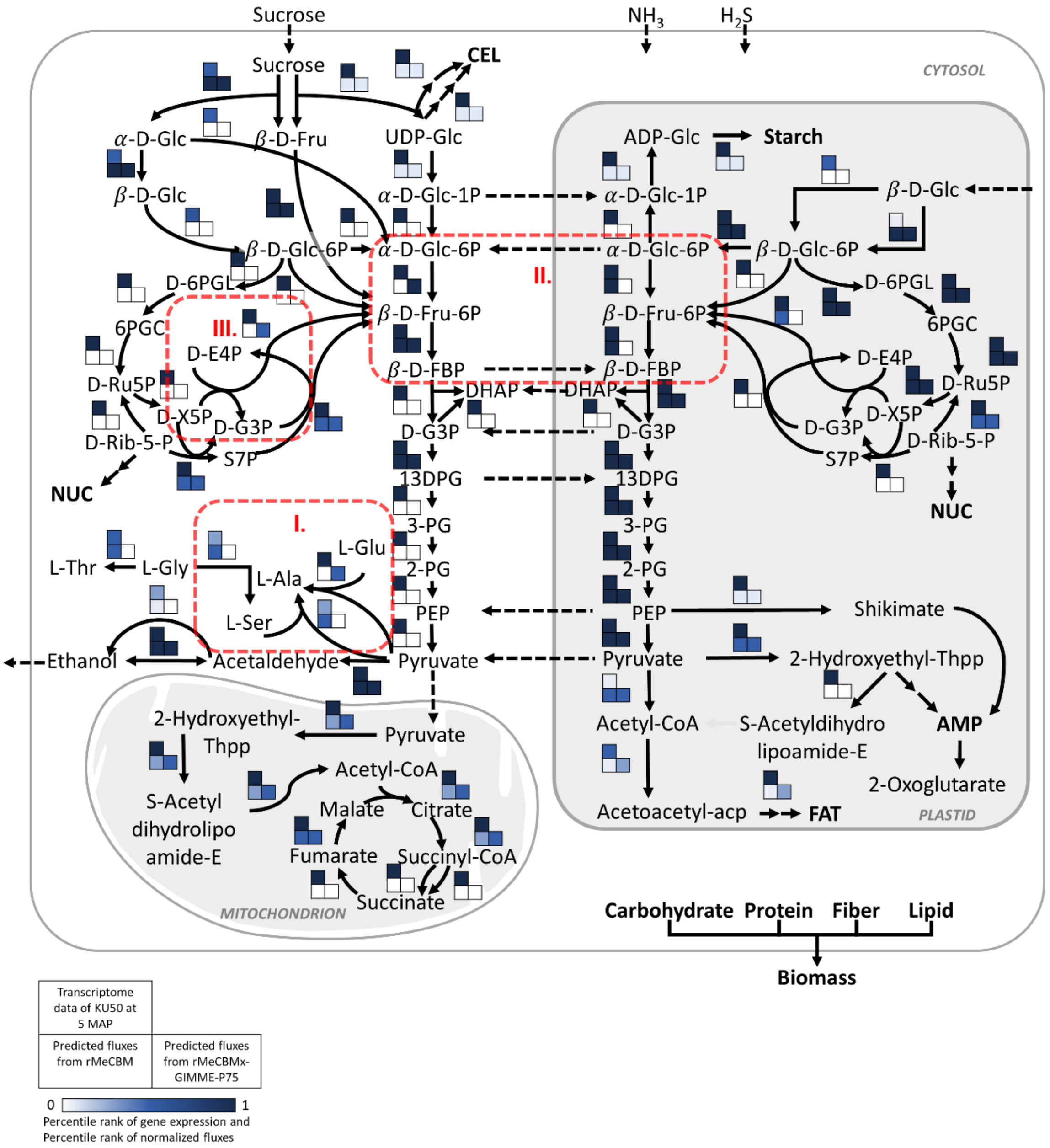

The rMeCBMx-GIMME-P25 and rMeCBMx-GIMME-P75 showed similar prediction. They predicted the use of pyruvate-glutamate transaminase (EC 2.6.1.2; R00258 remark as I.) to synthesis alanine instead of serine-pyruvate transaminase (EC 2.6.1.51; R00585) (mark as I.). They are different in the prediction of the carbon precursors imported from the cytosol for utilization in the plastid. rMeCBMx-GIMME-P75 preferred beta-D-Fructose 1,6-bisphosphate (β-D-FBP) in the plastid as similar to rMeCBM model, while rMeCBMx-GIMME-P25 imported alpha-D-glucose-6-phosphate (α-D-Glc-6P) for the respiration pathway and biosynthesis of other biomass components (remarked as II.). Additionally, they are different in the use of a bypass reaction in non-oxidative PPP, R01830-the conversion of D-erythose-4-phosphate (D-E4P) and D-xylulose-5-phosphate (D-X5P) to D-glyceraldehyde-3-phosphate (D-G3P) and beta-D-fructose-6-phostphate (β-D-Fru-6P) by transketolase (EC 2.2.1.1) (remarked as III.). rMeCBMx-GIMME-P25 predicted R01830 in the plastid, whereas rMeCBMx-GIMME-P75 predicted it in cytosol.

Supplementary Fig. S4 Comparison of flux distribution from rMeCBM and rMeCBMx-EFlux with transcriptome data of KU50 cultivar at five-month-old plant[1].

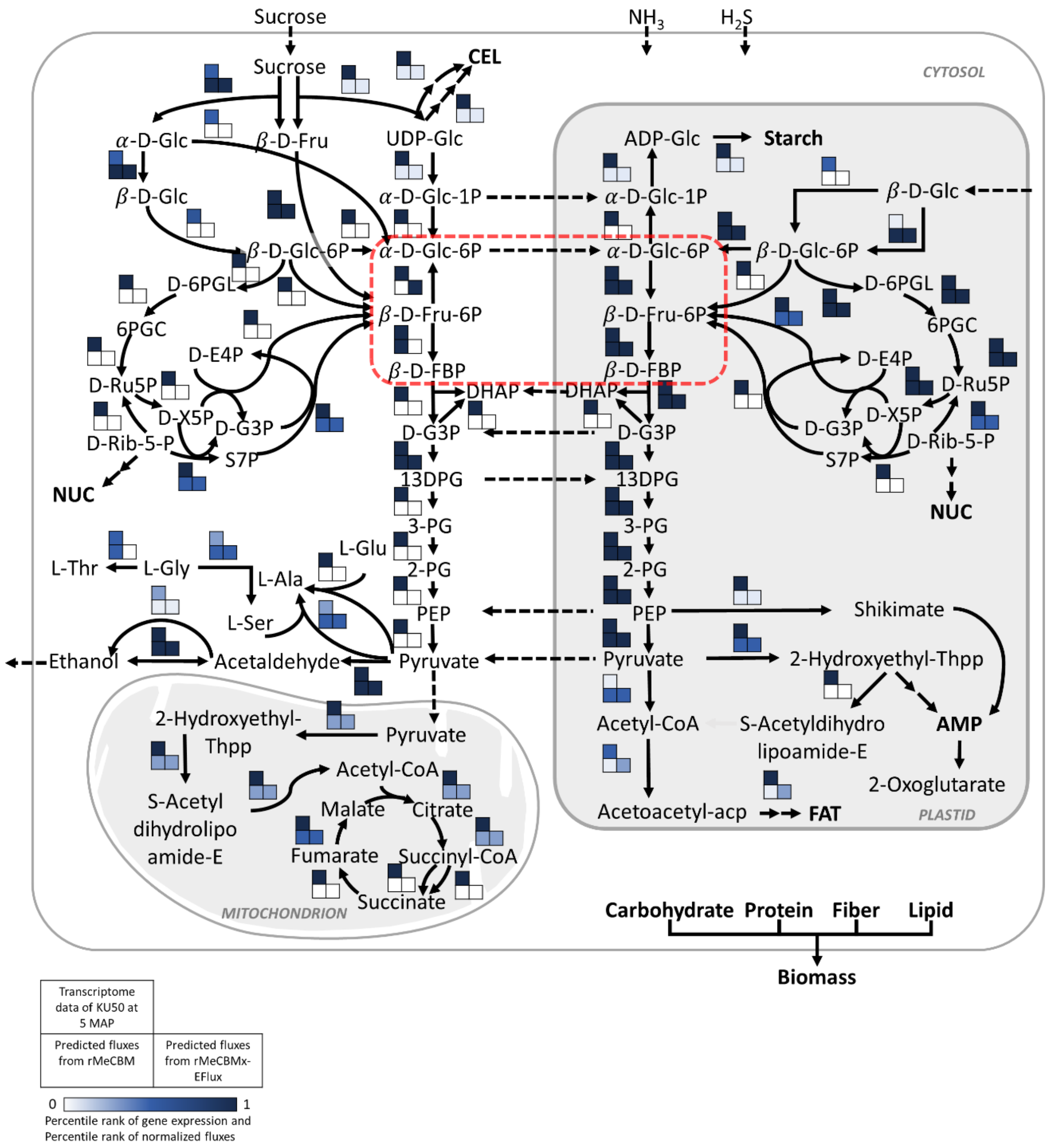

rMeCBMx-EFlux transported α-D-Glc-6P into plastid for the respiration pathway and biosynthesis of other biomass components instead of β-D-FBP.

Supplementary Fig. S5 Comparison of flux distribution from rMeCBM and rMeCBMx-HPCOF with transcriptome data of cultivar KU50 at five months old plant[1].

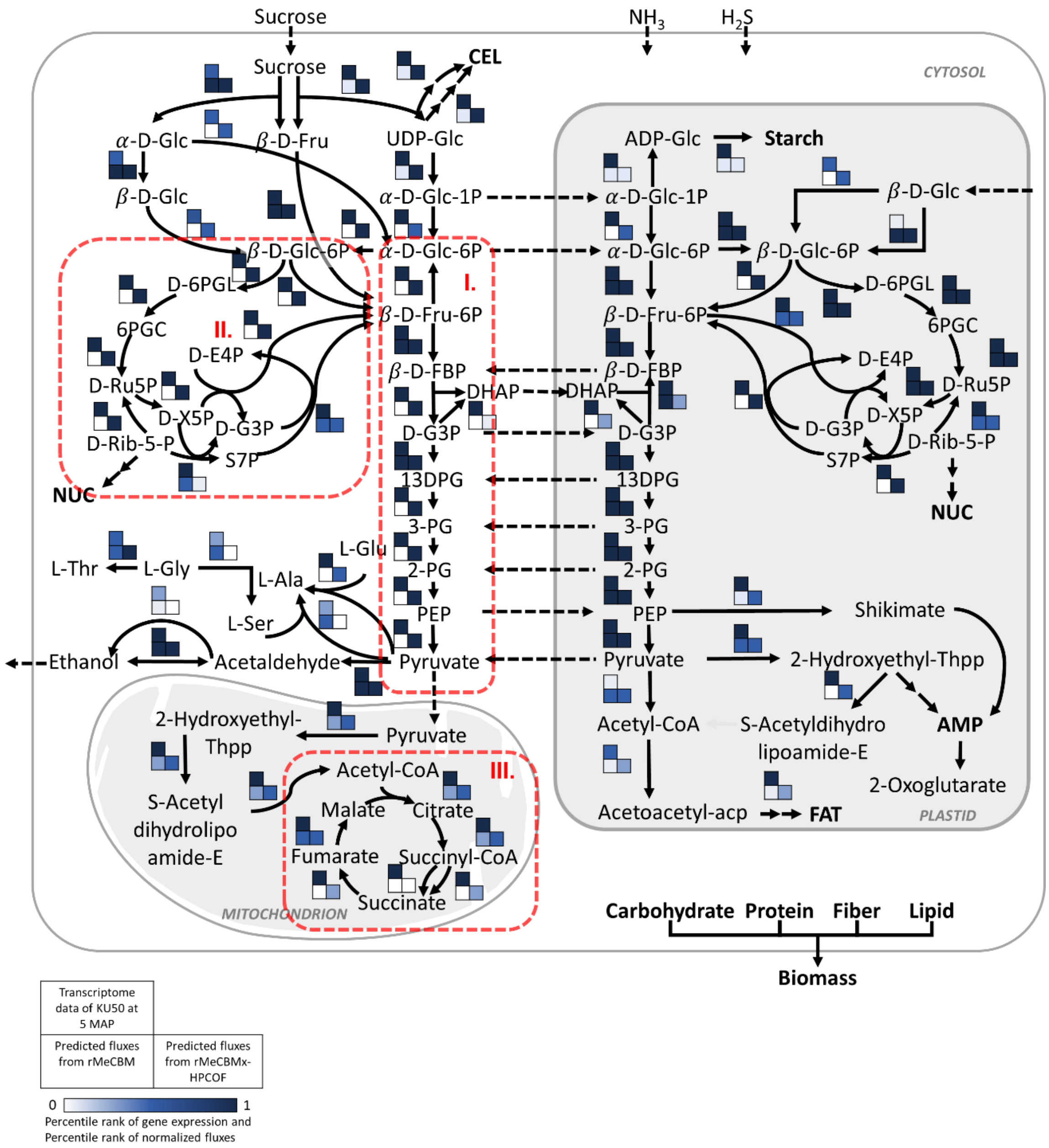

rMeCBMx-HPCOF was the only model that could capture the conversion of sugar phosphate to pyruvate through glycolysis (I.), the pentose phosphate pathway occurring in both cytosol and plastid (II.), and the full cycle of mitochondrial TCA (III.).

Supplementary Fig. S6 GO analysis of expressed genes in each representative scenario

A. The comparison of GO enrichment from total genes (> 0th percentile of gene expression) in three scenarios

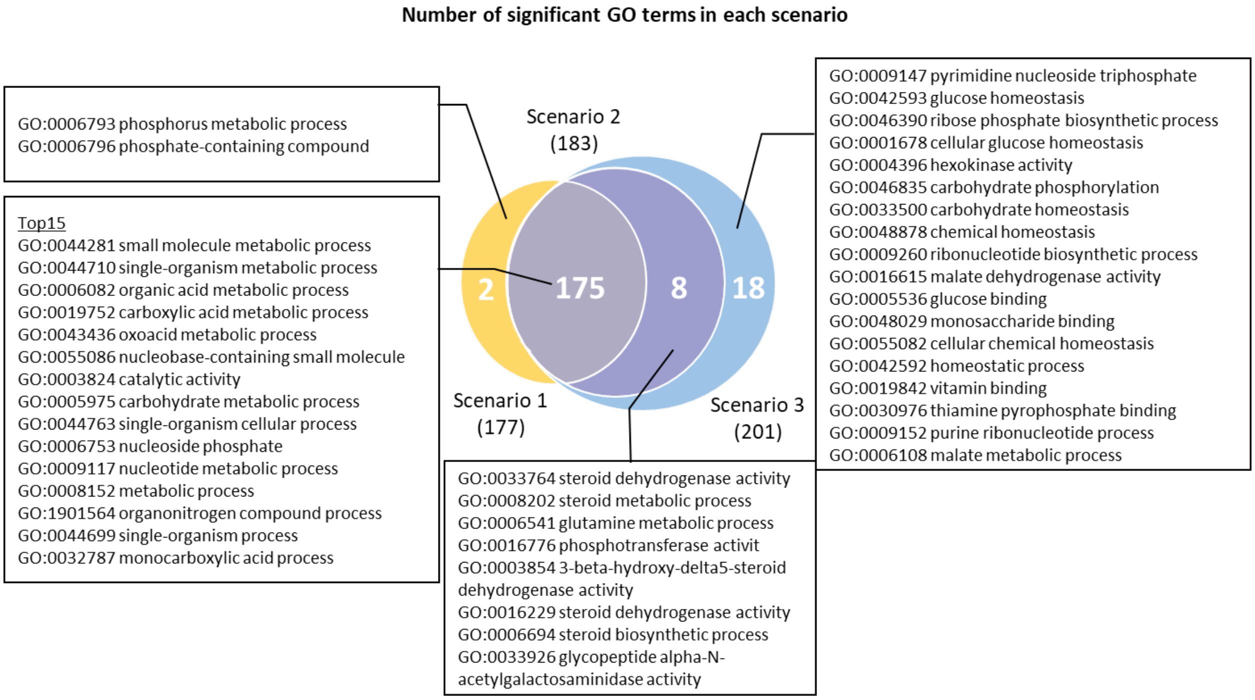

B. The comparison of GO enrichment from total genes (> 25th percentile of gene expression) in three scenarios

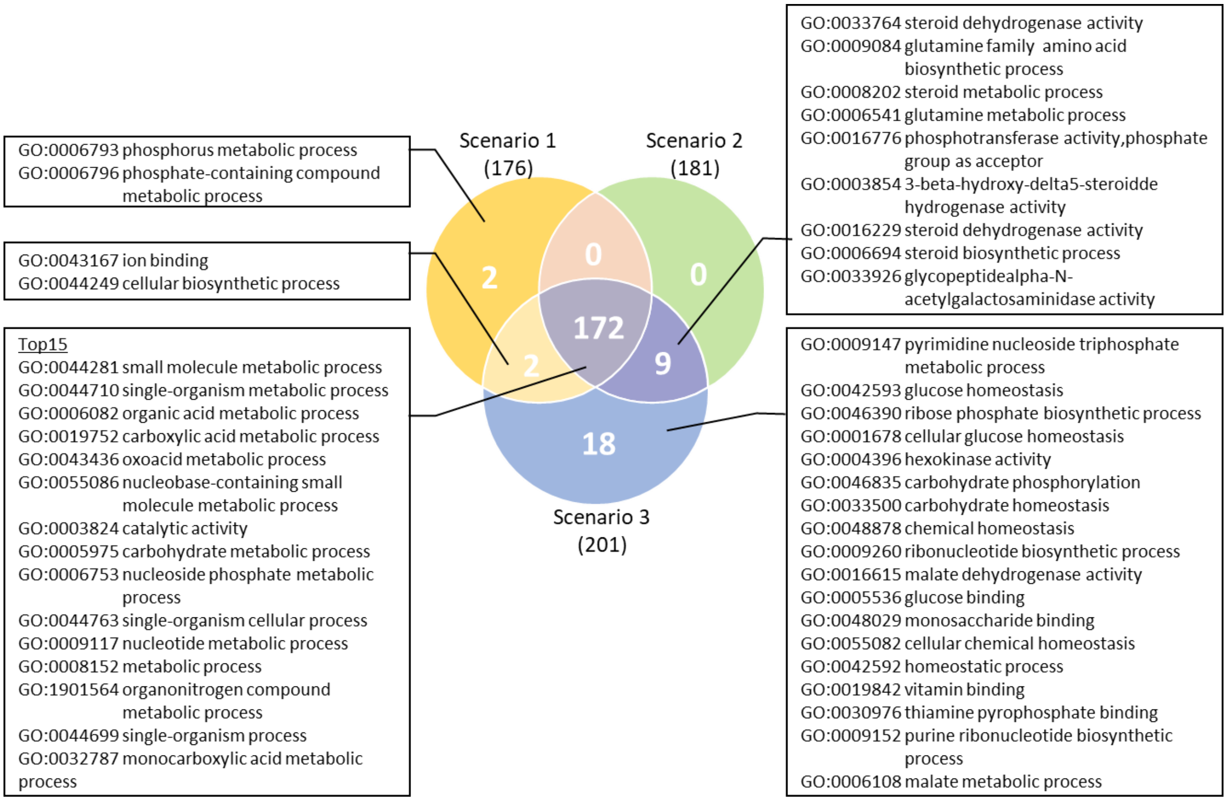

C. The comparison of GO enrichment from total genes (> 75th percentile of gene expression) in three scenarios

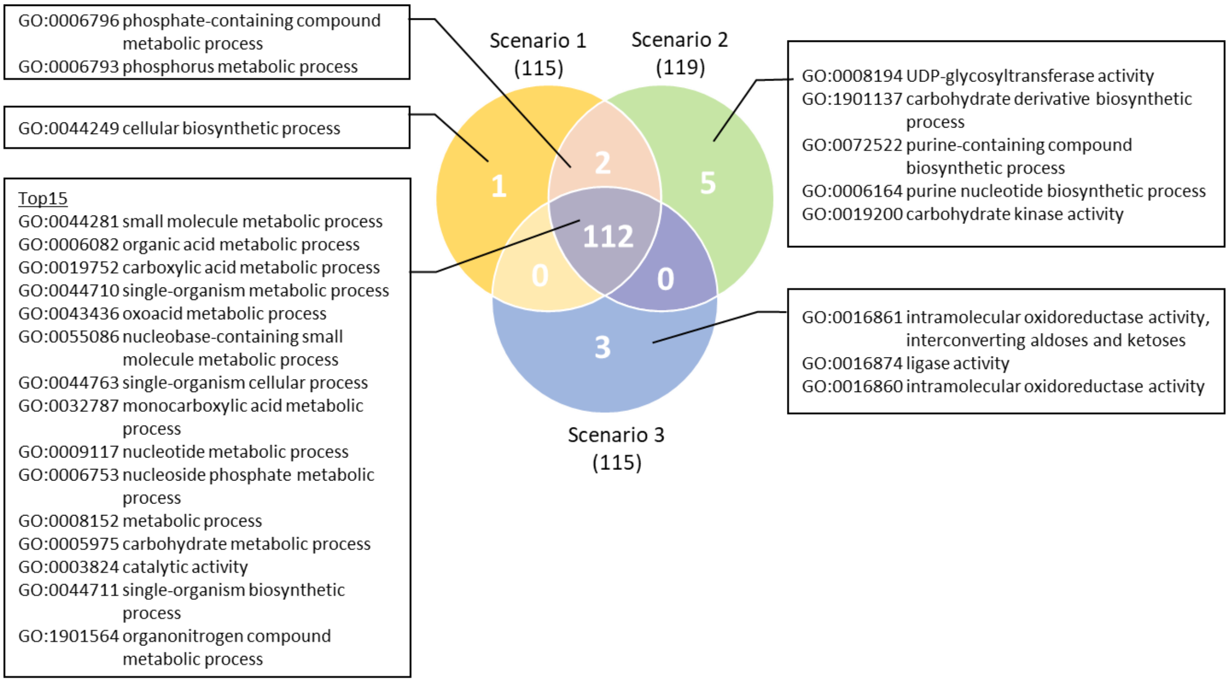

Supplementary Fig S7 The qualitative analysis of model performance based on three algorithms at varied thresholds of expressed genes from transcriptome data. (A) The 25th (low-rank) percentile of enzymatic gene expression in the model. (B) The 75th (high-rank) percentile of enzymatic gene expression in the model

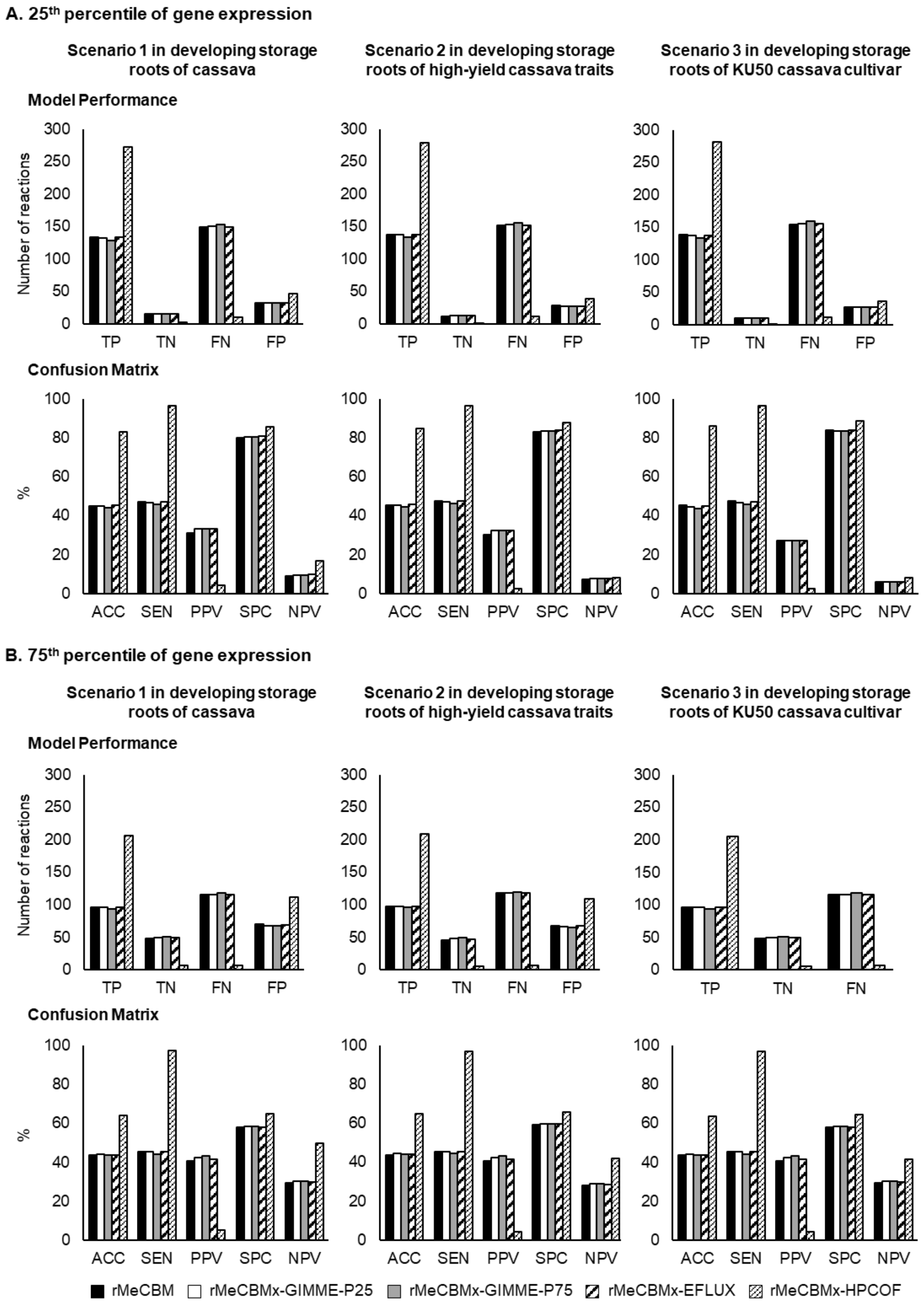

Supplementary Fig S8 Model validation using CMC9 physiological data, A: the harvest index of CMC9 and KU50 cultivars, B: comparison of predicted growth rate of CMC9 from rMeCBM and rMeCBMx-HPCOF with the measured growth rate

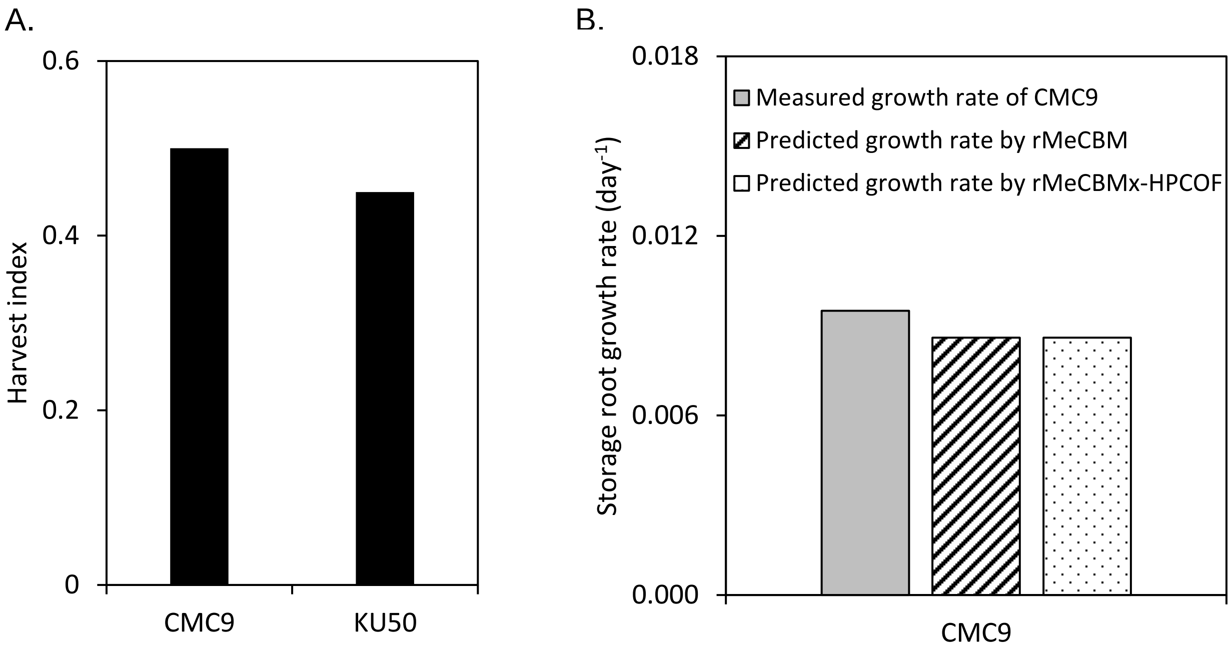

Supplementary Fig. S9 Sensitivity analysis of S_{GAM} to predicted growth rate of the rMeCBM model. Empty triangle and filled triangle represented S_{GAM} requirement of rMeCBM and rMeCBMx-HPCOF, respectively. Dotted lines lay out the percentage error of model simulated to measured storage roots growth rate

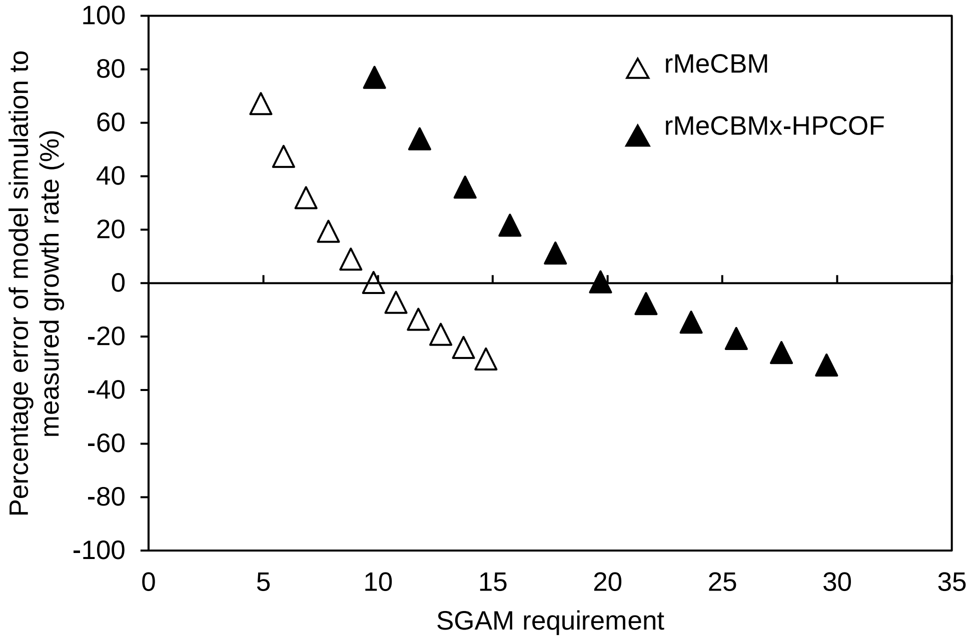

Supplementary Fig. S10 Cumulative frequency of supporting datasets from transcriptome data (A), proteome data (B), and metabolome data (C)

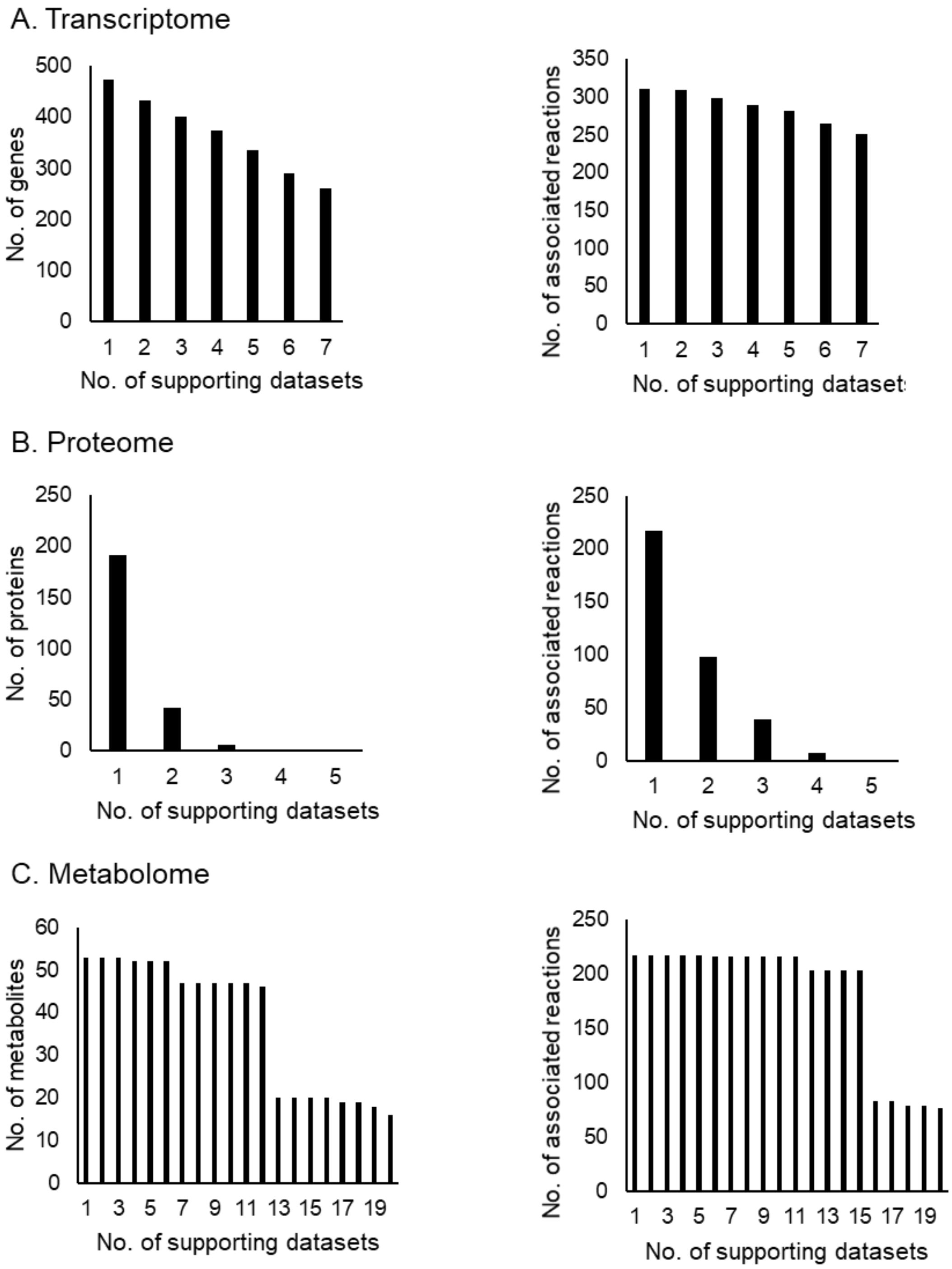

Supplementary Fig. S11 Comparison of active reactions from transcriptome, proteome, and metabolome with predicted fluxes from each model

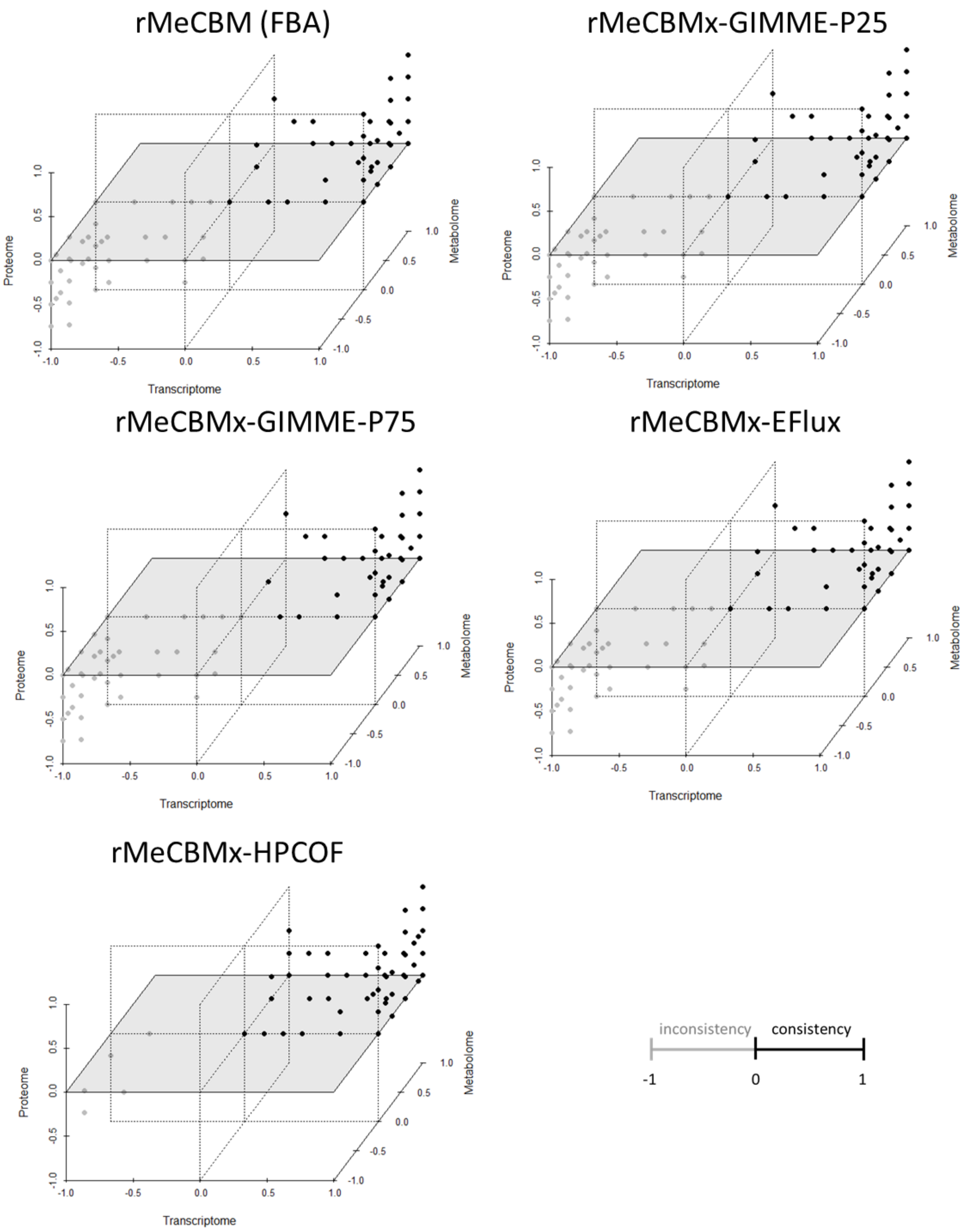

Supplement: Supplementary file 2 — Supplementary Figures. [file 41598_2021_88129_MOESM2_ESM.pdf]
