# Supplementary material for: Transcriptome integrated metabolic modeling of carbon assimilation underlying storage root development in cassava
Source: Sci Rep. 2021 Apr 22;11:8758. doi: 10.1038/s41598-021-88129-3 (PMC8062692; doi:10.1038/s41598-021-88129-3)
Supplement: Supplementary file 3 — Supplementary Tables. [file 41598_2021_88129_MOESM3_ESM.pdf]

Transcriptome integrated metabolic modeling of carbon assimilation underlying storage root development in cassava

Ratchaprapa Kamsen[1], **Saowalak Kalapanulak**[1,2], **Porntip Chiewchankaset**[2], **and Treenut Saithong**[1,2,*]

[1]Bioinformatics and Systems Biology Program, School of Bioresources and Technology and School of Information Technology, King Mongkut's University of Technology Thonburi (Bang Khun Thian), Bangkok, 10150, Thailand.
[2]Center for Agricultural Systems Biology, Systems Biology and Bioinformatics Research Group, Pilot Plant Development and Training Institute, King Mongkut's University of Technology Thonburi (Bang Khun Thian), Bangkok, 10150 Thailand
[*]corresponding author; e-mail: treenut.sai@kmutt.ac.th

Supplementary Table S1 The detail of available transcriptome data of cassava storage roots

No.	Datasets	Cassava cultivars	Age of plants (DAP)*	Biological replicates	Mapping rate (%)**	No. of expressed genes		References
						No. of expressed genes[a] (33,033)	No. of expressed metabolic genes in rMeCBM model[a] (762)	
1	Wang2014_W14-75	W14	75	-	86%	26,944	737	Wang *et al.*[1]
2	Wang2014_KU50-75	KU50	75	-	93%	27,201	731	Wang *et al.*[1]
3	Wang2014_KU50-120	KU50	120	-	93%	26,851	734	Wang *et al.*[1]
4	Wang2014_KU50-150	KU50	150	-	93%	28,247	724	Wang *et al.*[1]
5	Wang2014_Arg7-75	Arg7	75	-	89%	28,014	731	Wang *et al.*[1]
6	Wang2014_Arg7-120	Arg7	120	-	92%	27,846	732	Wang *et al.*[1]
7	Wang2014_Arg7-150	Arg7	150	-	92%	27,583	745	Wang *et al.*[1]
8	Wilson2017_TME-90	TME204	90	2	90%	23,188	731	Wilson *et al.*[2]

* DAP is days after planting

** Mapping rate is percentage of raw reads mapping to cassava AM560 genome (Bredeson *et al.*[3])

[a] Expressed genes were defined based on (a) a number of mapped reads are more than 5 reads and (b) gene expression level is greater than zero FPKM. Number in parenthesis is total genes in cassava AM560 genome (33,033) and total genes in rMeCBM model (762 genes). respectively

Supplementary Table S2 The characteristics of proteome data in developing cassava storage roots, including number of expressed proteins, and associated reactions

No.	Datasets	Characteristics				Proteins				Reactions	References
		Cassava cultivars	Age of plants (MAP)*	Protein identification method	No. of datasets	No. of identified proteins[a] (unique protein IDs)	No. of annotated proteins in cassava genome V.4.1[a]	No. of annotated proteins in cassava genome V.6.1[b]	No. of annotated proteins in rMeCBM[c]	No. of reactions in rMeCBM related to annotated proteins[d]	
1	Sheffield2006	-	3	QTOF-MS/MS	1	42	-	36	8	29	Sheffield *et al.*[5]
2	Li2010	SC8	3	LC-ESI-MS/MS	1	155	-	210	48	83	Li *et al.*[6]
3	Owiti2011	-	-	LC-MAIDI-MS	1	949	-	39	5	7	Owiti *et al.*[7]
4	Vanderschuren2014	TMS60444	10	TP3Q-MS/MS	1	-	1194	1031	166	203	Vanderschuren *et al.*[8]
5	Naconsie2015	KU50	3	LC-MS/MS	1	95	-	61	13	39	Naconsie *et al.*[9]
				Total (unique)	5	-	-	1180	192	217	

[*] MAP is months after planting

[a] data was collected from literatures which belong to cassava storage roots at control condition.

[b] annotated proteins in cassava genome V.6.1 were identified if the identity percentage ≥ 60, coverage percentage ≥ 80 and e-value ≤ 10^{-10} using Blastp and proteins from all datasets as the query.

[c] the total number of proteins based on cassava genome V.6.1 in rMeCBM model is 852 proteins

[d] the protein-related reactions in rMeCBM model consist of 330 reactions

Supplementary Table S3 The characteristics of metabolomic data in developing cassava storage roots including number of metabolites, and associated reactions

No.	Datasets	Characteristics					Metabolites		Reactions	References
		No. of cultivars	Age of plant (MAP)*	Cultivation system	Protein identification method	No. of datasets	No. of identified metabolites[a]	No. of matching metabolites in rMeCBM[b]	No. of reactions in rMeCBM related to metabolites[c]	
1	Drapal2020_ Field	8[d]	10	Field	GC-MS	8	93	25	106	Drapal *et al.*[10]
2	Obata2020_Field	6[e]	4	Field	GC-MS	6	126	53	217	Obata *et al.*[11]
3	Obata2020_Greenhouse	6[e]	3	Greenhouse	GC-MS	6	126	53	217	Obata *et al.*[11]
					Total (unique)	20	158	53	217	

[*] MAP is months after planting

[a] data was collected from literatures which belong to cassava storage roots at control condition.

[b] the total number of metabolites in rMeCBM model is 393 metabolites

[c] the protein-related reactions in rMeCBM model consist of 330 reactions

[d] Eight cassava cultivars: BRA1A, COL22, COL638, CUB23, PER183, PER283, PER483, and PER583

[e] Six cassava cultivars: IITA-TMS-IBA980581, IITA-TMS-IBA980002, IITA-TMS-IBA30572, IITA-TMS-IBA011412, TMEB693, and TMEB419

Supplementary Table S4 Simulation of storage root growth rate through FBA and transcriptome-integrated metabolic models of cassava storage roots

Algorithms	S_{GAM} ($mmol_{ATP}$ gDW^{-1}_{SRs})	Storage root growth rate (day^{-1})		ε(%)
		Prediction	Experiment*	
rMeCBM (FBA)	9.8	0.0090	0.0090	0
rMeCBMx-GIMME-P25	9.8	0.0090	0.0090	0
rMeCBMx-GIMME-P50	9.8	0.0090	0.0090	0
rMeCBMx-GIMME-P75	9.8	0.0090	0.0090	0
rMeCBMx-GIMME-P90	9.8	0.0090	0.0090	0
rMeCBMx-EFlux	9.8	0.0090	0.0090	0
rMeCBMx-HPCOF	19.7	0.0090	0.0090	0

ε was the percentage error of prediction to measured cassava storage root growth rate

* Measured growth rate from experiment was obtained from Chiewchankaset *et al*[4].

Supplementary Table S5 The expression-guided active metabolic reactions in carbon assimilation pathway of cassava storage roots inferred by using different thresholds

Models	Thresholds of gene expression in rMeCBM model	No. of expressed genes related in rMeCBM model	Total reactions (468 reactions)			Biochemical reactions with genes (330 reactions)			
			No. of active reactions based on a given threshold	No. of reactions with non-zero fluxes	No. of reactions with zero fluxes	No. of active reactions based on a given threshold	No. of reactions with non-zero fluxes	No. of reactions with zero fluxes	No. of recovered low gene expression reactions
rMeCBM (FBA)	-	731	468	251	217	330	163	167	-
rMeCBMx-GIMME-P25	25th percentile	518	460	251	217	325	161	169	2
rMeCBMx-GIMME-P50	50th percentile	345	421	254	214	283	166	164	22
rMeCBMx-GIMME-P75	75th percentile	173	339	249	219	201	158	172	62
rMeCBMx-GIMME-P90	90th percentile	70	253	249	219	115	159	171	112

Supplementary Table S6 Identification of expressed genes in each representative scenario based on coefficient of variance (CV). It assumed that the representative gene set denoted the expressed genes in charge of regulation under the scenario was considered if the variation in terms of CV between the datasets in scenario was less than 25 percent. The representative gene set was also categorized based on expression level, in percentile basis of individual datasets

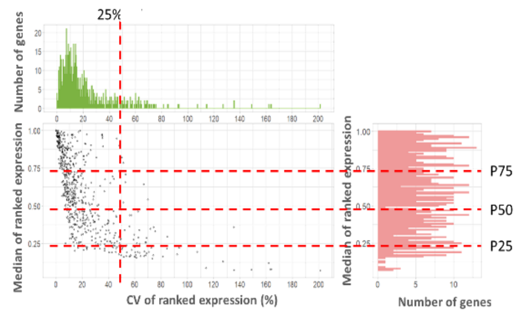

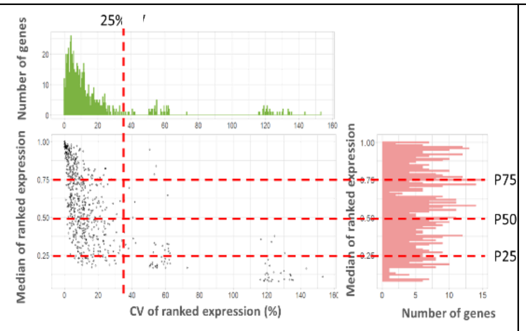

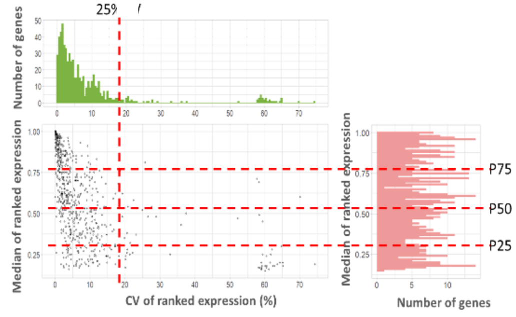

Threshold of gene expression levels	Scenario 1 in developing storage roots of cassava (all four cultivars)		Scenario 2 in developing storage roots of high-yield traits (KU50 and ARG7)		Scenario 3 in developing storage roots of KU50 cultivar	
	No. of expressed genes (763 genes)	No. of active reactions (330 reactions)	No. of expressed genes (763 genes)	No. of active reactions (330 reactions)	No. of expressed genes (763 genes)	No. of active reactions (330 reactions)
> The 0th percentile	472	330	545	330	589	330
> The 25th percentile	464	282	524	290	552	293
> The 50th percentile	354	268	369	270	379	266
> The 75th percentile	180	212	188	216	191	212

Supplementary Table S7 GO analysis of the expressed gene set in each representative scenario

	Scenario 1 in developing storage roots of cassava (all four cultivars)				Scenario 2 in developing storage roots of high-yield traits (KU50 and ARG7)				Scenario 3 in developing storage roots of KU50 cultivar			
	No. of genes	No. of genes with annotated GOs	% of genes with annotated GOs	No. of enriched GOs	No. of genes	No. of genes with annotated GOs	% of genes with annotated GOs	No. of enriched GOs	No. of genes	No. of genes with annotated GOs	% of genes with annotated GOs	No. of enriched GOs
> The 0th percentile	472	367	77.75	177	545	421	77.25	183	589	461	78.27	201
> The 25th percentile	464	363	78.23	176	524	409	78.05	181	552	439	79.53	201
> The 50th percentile	354	282	79.66	160	369	290	78.59	169	379	295	77.84	166
> The 75th percentile	180	145	80.56	115	188	152	80.85	119	191	152	79.58	115

References

1. Wang, W. *et al.* Cassava genome from a wild ancestor to cultivated varieties. *Nat. Commun.* **5**, (2014).

2. Wilson, M. C. *et al.* Rapid report Gene expression atlas for the food security crop cassava. (2017) doi:10.1111/nph.14443.

3. Bredeson, J. V *et al.* resource Sequencing wild and cultivated cassava and related species reveals extensive interspecific hybridization and genetic diversity. **34**, (2016).

4. Chiewchankaset, P., Siriwat, W. & Suksangpanomrung, M. Understanding carbon utilization routes between high and low starch-producing cultivars of cassava through Flux Balance Analysis. *Sci. Rep.* 1–15 (2019) doi:10.1038/s41598-019-39920-w.

5. Sheffield, J., Taylor, N., Fauquet, C. & Chen, S. The cassava (Manihot esculenta Crantz) root proteome : 1588–1598 (2006) doi:10.1002/pmic.200500503.

6. Li, K. *et al.* Proteome characterization of cassava (Manihot esculenta Crantz) somatic embryos , plantlets and tuberous roots. 1–12 (2010).

7. Owiti, J. *et al.* iTRAQ-based analysis of changes in the cassava root proteome reveals pathways associated with post-harvest physiological deterioration. 145–156 (2011) doi:10.1111/j.1365-313X.2011.04582.x.

8. Vanderschuren, H. Large-Scale Proteomics of the Cassava Storage Root and Identification of a Target Gene to Reduce Postharvest Deterioration Large-Scale Proteomics of the Cassava Storage Root and Identi fi cation of a Target Gene to Reduce Postharvest Deterioration. (2014) doi:10.1105/tpc.114.123927.

9. Naconsie, M., Lertpanyasampatha, M. & Viboonjun, U. Cassava root membrane proteome reveals activities during storage root maturation. *J. Plant Res.* (2015) doi:10.1007/s10265-015-0761-4.

10. Drapal, M., Ovalle Rivera, T. M., Becerra Lopez-Lavalle, L. A. & Fraser, P. D. Exploring the chemotypes underlying important agronomic and consumer traits in cassava (Manihot esculenta crantz). *J. Plant Physiol.* **251**, 153206 (2020).

11. Obata, T. *et al.* Metabolic profiles of six African cultivars of cassava (Manihot esculenta Crantz) highlight bottlenecks of root yield. *Plant J.* 1202–1219 (2020) doi:10.1111/tpj.14693.
